# Tumor Organoids Grown in Mixed-Composition Hydrogels Recapitulate the Plasticity of Pancreatic Cancers

**DOI:** 10.3390/gels11070562

**Published:** 2025-07-21

**Authors:** Ioritz Sorzabal-Bellido, Xabier Morales, Iván Cortés-Domínguez, Maider Esparza, Lucía Grande, Pedro Castillo, Silvia Larumbe, María Monteserín, Shruthi Narayanan, Mariano Ponz-Sarvise, Silve Vicent, Carlos Ortiz-de-Solórzano

**Affiliations:** 1 Program in Biomedical Engineering, CIMA Universidad de Navarra and Cancer Center Clínica Universidad de Navarra (CCUN), 31008 Pamplona, Spain; ioritzsorzabal@gmail.com (I.S.-B.); icortesd@unav.es (I.C.-D.); pcastillor@unav.es (P.C.); 2 Imaging Platform, CIMA Universidad de Navarra, 31008 Pamplona, Spain; xmorales@unav.es (X.M.); maideresparza@unav.es (M.E.); lgrande@unav.es (L.G.); 3 Department of Physics and Applied Mathematics, University of Navarra, 31008 Pamplona, Spain; 4 Navarra Health Institute (IDISNA), 31008 Pamplona, Spain; mponz@unav.es (M.P.-S.); silvevicent@unav.es (S.V.); 5 Centre for Surface Engineering and Advanced Materials, Asociación de la Industria Navarra (AIN), 31191 Cordovilla, Spain; slarumbe@gcosmos.com (S.L.); mmonteserin@ain.es (M.M.); 6 Program in Solid Tumors, CIMA Universidad de Navarra and Cancer Center Clínica Universidad de Navarra (CCUN), 31008 Pamplona, Spain; snarayanan@unav.es; 7 Department of Medical Oncology, and Program in Solid Tumors, CIMA Universidad de Navarra and Cancer Center Clínica Universidad de Navarra (CCUN), 31008 Pamplona, Spain; 8 Centro de Investigación Biomédica en Red de Cáncer (CIBERONC), 28029 Madrid, Spain; 9 Department of Pathology, Anatomy and Physiology, University of Navarra, 31008 Pamplona, Spain; 10 Department of Biomedical Engineering, TECNUN, University of Navarra, 20018 San Sebastián, Spain

**Keywords:** PDAC, tumor-derived organoids, biomimetic hydrogels, extracellular matrix (ECM), mechanobiology, plasticity, epithelial-to-mesenchymal transition (EMT), remodeling, metabolism, resistance

## Abstract

Pancreatic ductal adenocarcinoma (PDAC) tumors exhibit pronounced phenotypic plasticity, alternating between a treatment-sensitive classical phenotype and a more aggressive basal-like state associated with drug resistance and poor prognosis. The frequent coexistence of these phenotypes complicates patient stratification and the selection of effective therapies. Tumor-derived organoids are valuable tools for drug screening; however, their clinical relevance relies on how accurately they recapitulate the phenotypic and functional characteristics of the original tumors. In this study, we present a quantitative analysis of how hydrogel composition influences the phenotype, tissue remodeling, metabolism, and drug resistance of PDAC organoids. Organoids were cultured within three types of hydrogels: Matrigel, collagen-I, and a mixture of collagen-I and Matrigel. Our results demonstrate that: (i) PDAC organoids grown in Matrigel exhibit a classical phenotype, with metabolic and drug response profiles similar to those of low-physiological two-dimensional cultures; (ii) Organoids grown in collagen-containing hydrogels, particularly those in collagen-Matrigel composites, faithfully recapitulate basal-like tumors, characterized by epithelial-to-mesenchymal transition, tissue remodeling, metabolic activity, and drug resistance; (iii) TGFβ induces an exacerbated, highly invasive basal-like phenotype. Summarizing, our findings highlight the importance of 3D hydrogel composition in modulating PDAC organoid phenotype and behavior and suggest collagen-Matrigel hydrogels as the most suitable matrix for modeling PDAC biology.

## 1. Introduction

Pancreatic cancer is the third leading cause of cancer-related mortality, with a five-year survival rate below 13%. Pancreatic ductal adenocarcinoma (PDAC), which accounts for approximately 90% of all pancreatic cancer cases [1], is projected to become one of the leading causes of cancer-related deaths [2], partly due to the limited efficacy of current chemotherapy treatments [3,4]. A contributing factor is the highly desmoplastic nature of PDAC tumors, whose extracellular matrix (ECM) comprises a dense network of collagens I, III, and IV, laminin and fibronectin. This rigid tumor microenvironment (TME) not only promotes tumor cell invasiveness but also acts as a physical barrier that impedes drug delivery [5]. Recent studies have identified two major, and often co-existent clinical PDAC subtypes [6], the classical subtype, associated with a more favorable prognosis, and the basal-like subtype, which correlates with drug resistance and poor clinical outcomes [7]. These phenotypes are governed by the interplay of both tumor-intrinsic and stromal gene expression programs. Consequently, accurate modeling of PDAC biology requires advanced models that accurately recapitulate the complex three-dimensional (3D) mechanobiology of the tumor, including contributions from both tumor cells and the ECM [8,9]. Animal models remain a valuable alternative for studying PDAC. However, they are costly, offer limited experimental flexibility, and pose significant ethical concerns [10,11,12]. In this context, organoid models, derived from tumor samples and grown within 3D ECM-mimetic hydrogels, have emerged as a promising alternative [13]. Tumor organoids retain key genetic and proteomic features of the original tumors, including their unique intra- and inter-tumoral heterogeneity, and preserve physiologically relevant cell-cell and cell-ECM interactions. Patient-derived tumor organoids (PDTOs), established directly from clinical samples, hold significant translational potential [14,15], although their use is limited by issues of availability and reproducibility. In contrast, organoids derived from murine cancer models, representative of the human disease [16] offer enhanced availability and experimental control [17,18]. These models may help bridge the translational gap posed by the scarcity of PDTOs and serve as ideal platforms to investigate tumor-ECM interactions in a physiologically relevant 3D context [19].

The ECM [20] is a 3D network composed of fibrous proteins, soluble factors, bioactive molecules, and mechanotransduction cues [21,22]. In healthy tissues, ECM homeostasis is tightly regulated. However, during cancer progression, this balance is disrupted, leading to dynamic interactions between tumor cells and the surrounding ECM that alter its biomechanical properties [21,23,24]. These changes contribute to tumor progression by enhancing cell invasiveness [25], and promoting malignant transformation through the acquisition of mesenchymal-like traits [26], a process that in PDAC is strongly driven by TGFβ signaling [27]. These insights highlight the crucial role of ECM mechanobiology in tumor development and drug resistance, and emphasize the importance of carefully considering the mechanobiology and biochemical properties of the scaffolding components when developing physiologically relevant organoid models. Despite this, such considerations are often unreported or overlooked in the literature [22,28,29].

Matrigel remains the most used scaffolding material for 3D organoid cultures. It is rich in ECM components, including collagen-IV, laminin and entactin. However, its use presents limitations, including batch-to-batch variability and low mechanical stiffness, which compromise experimental reproducibility and limit the physiological relevance of the resulting models. Synthetic hydrogels [30], composed on materials such as polyethylene-glycol, polycaprolactone, poly (lactic-co-glycolic) acid, or poly (isocyanide) derivatives, offer optimal, tunable biomechanical properties [31], making them ideal for dissecting the role of specific biomechanical cues in tumor growth. However, these materials lack intrinsic bioactivity, thus requiring bio-functionalization with cell-binding motifs and supplementation with complex mixtures of growth factors. Natural polymer-based hydrogels, derived from proteins such as collagen-I and fibrin or polysaccharides such as hyaluronic acid and alginate, are inherently biofunctional but often display suboptimal rheological properties. Achieving a balance between pre-polymer solution manipulability and post-polymerization structural integrity remains a technical challenge [29].

We hypothesize that hydrogels of mixed composition, combining natural polymers with Matrigel, best mimic the ECM of PDAC tumors and enable recapitulation of the phenotypic plasticity observed in the clinical samples, both in vivo and in vitro. To validate this, we conducted a comprehensive analysis using biomimetic hydrogels composed of Matrigel, collagen-I, or a collagen-Matrigel mixture [25,32] to support the growth of PDAC organoids. Collagen-I contributes to bio-functionality and modulates matrix stiffness, while Matrigel supplies a rich biochemical environment similar to the tumor ECM and regulates the viscous properties of the scaffold [33,34]. We evaluated the impact of hydrogel composition on a range of phenotypic descriptors, including “early-stage organoid seeds” (EOSs) migration, mature organoid morphology, lattice remodeling, metabolic activity, and the expression of markers of epithelial-to-mesenchymal transition (EMT) and clinical subtype under regular media or TGFβ conditioning. Furthermore, we evaluated how the biomechanical properties of the hydrogels influenced the response of PDAC organoids to Gemcitabine (Gem), both in vitro and in vivo using an immunocompetent murine model.

Our findings reveal substantial differences in organoid phenotype depending on hydrogel composition, underscoring the pivotal role of ECM mechanobiology in modulating the chemoresistance of PDAC organoids. These results provide valuable insights for researchers employing organoid models in drug discovery and translational pancreatic cancer research.

## 2. Results

### 2.1. Collagen-I Content Modulates Hydrogel Micro-Architecture and Viscoelastic Behavior

**Figure 1:**
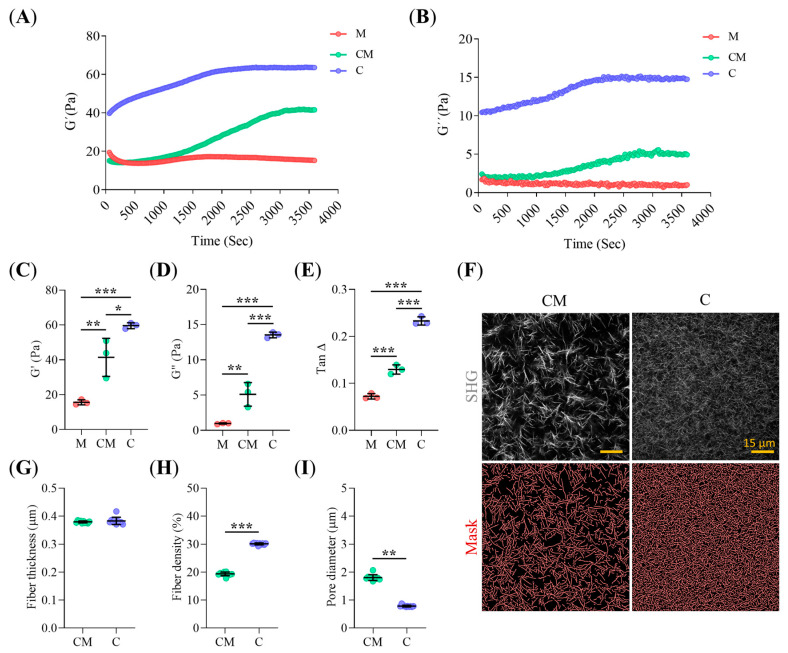
Hydrogel rheological and mechanical characterization. (**A**,**B**) Storage (G′, Pa) and loss (G″, Pa) moduli profiles during oscillatory time sweep assays for M, CM, and C hydrogels. (*n* = 3 biological replicates); (**C**–**E**) Quantification of storage (G′) and loss (G″) moduli, and TanΔ of M, CM, and C hydrogels after gel polymerization, extracted from oscillatory time sweep assays. (*n* = 3 biological replicates); (**F**) Representative MIP projection of SHG CLSM images of collagen-I fiber lattice in CM and C Hydrogels. (Collagen-I segmentation masks in Red). Scale Bars: 15 µm; (**G**–**I**) Quantitative analysis of collagen-I fibre lattice morphology, including fibre thickness (μm), fibre density (%), and pore diameter (μm), respectively, extracted from images in panel F. (*n* = 10 images from 3 biological replicates). Error bars: mean ± SD; ***, *p* < 0.001, **, *p* < 0.01, *, *p* < 0.05. One-way ANOVA (**C**–**E**). Mann-Whitney test (**G**–**I**).

We used three hydrogels: M (Matrigel 4 mg/mL), C (collagen-I 4 mg/mL) and CM (mixture of collagen-I 2 mg/mL and Matrigel 2 mg/mL). We first characterized their mechanical properties and lattice microstructure. The rigidity and elastic components of the hydrogels, measured by the storage modulus (G′), increased with increasing collagen-I and decreasing Matrigel content (Figure 1A,C). A similar trend was observed for viscosity, as indicated by the loss modulus (G″) (Figure 1B,D). The G″/G′ ratio (Tan∆) was close to zero in all three hydrogels, consistent with a predominantly solid, elastic behavior (Figure 1E). However, the presence of collagen increased Tan∆, reflecting a greater contribution of the viscous component in collagen-containing hydrogels. Second Harmonic Generation (SHG) imaging of collagen-I fiber distribution in C and CM hydrogels (Figure 1F) revealed no significant differences in collagen-I fiber thickness (Figure 1G). However, mixed CM hydrogels exhibited larger pore size diameter (Figure 1H) and lower fiber density (Figure 1I) compared to C hydrogels.

### 2.2. Collagen-Related ECM Stiffening and TFGβ Promote Pro-Invasive Traits in Early-Stage PDAC93 Organoid Seeds

**Figure 2:**
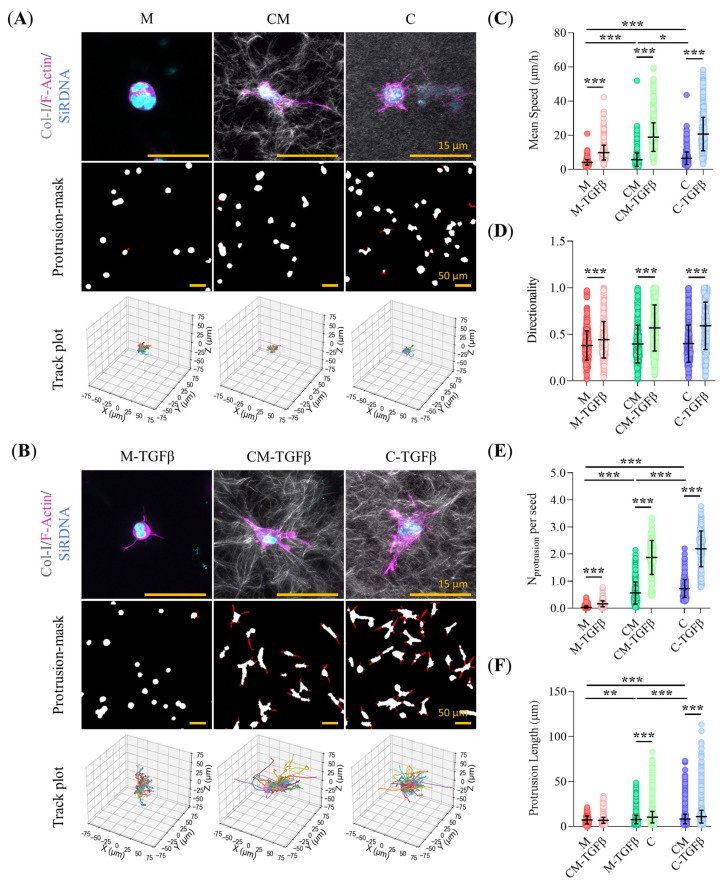
Analysis of “Early Organoid Seed” (EOS) migration dynamics. (**A**,**B**) Representative Z-stack projections of 3D CLSM images of EOSs within M, CM, and C hydrogels, under regular media or TGFβ conditioning. Staining: F-Actin (magenta), nuclei (SiRDNA, cyan), and the collagen-I lattice (SHG). Protrusion masks (in red) highlight cytoplasm extensions. The 3D track plots illustrate the length and directness of the EOSs trajectories. Scale bars: 15 µm for CLSM images (**upper panel**) and 50 µm for protrusion masks (**bottom panel**); (**C**–**F**) Quantitative analysis of invasive descriptors extracted from panels A and B included mean speed (μm/h), directionality, and the number and length of protrusions (μm), respectively. (*n* = at least 400 EOSs from 3 biological replicates) (**C**,**D**) (*n* = at least 250 protrusions from 3 biological replicates) (**E**,**F**). Error bars: mean ± SD; ***, *p* < 0.001, **, *p* < 0.01, *, *p* < 0.05. Wilcoxon rank-sum test and Mann-Whitney test (**C**–**F**).

Prior to organoid formation, PDAC93 cells actively migrated through the hydrogel, establishing cell-to-cell contacts and forming “early organoid seeds” (EOSs). We analyzed the migration of EOSs using time-lapse microscopy. Representative EOSs images and corresponding 3D track plots are shown in Figure 2A,B. We observed a significant increase in migration speed (Figure 2C), as well as in the number and length of EOSs cell protrusions (Figure 2E,F), when comparing hydrogels M and CM, a less intense but still significant increase observed between CM and C hydrogels. TGFβ conditioning further enhanced EOSs migration speed and the number and length of cell protrusions across all three hydrogels, especially in collagen-I containing hydrogels. No significant differences in migration directionality were observed among the different hydrogel conditions, as EOSs follow random migration patterns regardless of hydrogel composition (Figure 2D).

### 2.3. ECM Biomechanical Properties and TGFβ Modulate the Morphology of Mature PDAC93 Organoids

**Figure 3:**
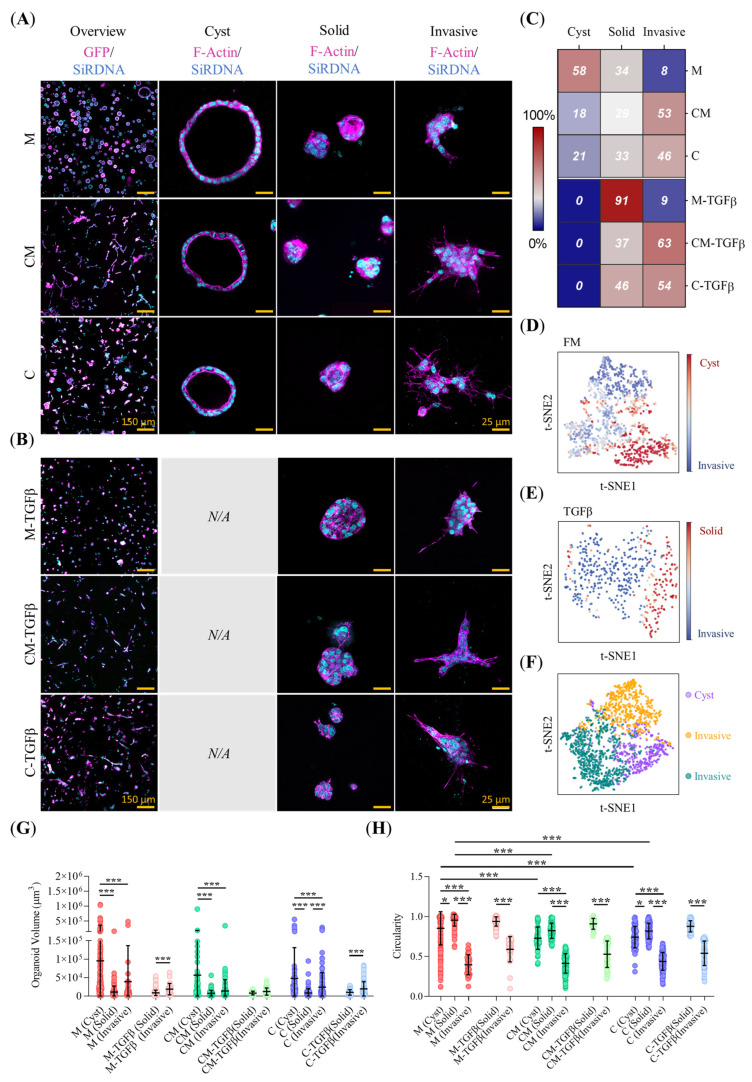
Collagen-I and TGFβ conditioning modulate the morphological plasticity of mature PDAC93 organoids. (**A**,**B**) Representative Z-stacks projections of 3D CLSM images of GFP-PDAC93 organoids grown within M, CM and C hydrogels, under regular media or TGFβ conditioning. Staining: cytoplasm (endogenous-GFP, magenta) and nuclei (SiRDNA, cyan). “*N*/*A*” indicates the absence of representative images for a given organoid class under those specific conditions, based on microscope observations and SVM classifier data. Scale bars: 150 µm for low-magnification images (**left panel**) and 25 µm for high-magnification images (**right panel**); (**C**) Abundance heat-map of PDAC93 organoid phenotypes. (*n* = 3 biological replicates); (**D**,**E**) t-SNE projection showing the morphological class distribution of different PDAC93 organoids under regular media or TGFβ conditioning, respectively, combining data from M, CM, and C hydrogels. The scale bar represents the classification probability as Cyst, Solid, or Invasive; (**F**) t-SNE projection showing the distribution of the three morphological classes identified by the SVM classifier, combining data from all hydrogels and media conditions; (**G**,**H**) Quantification of the morphological features used in the SVM classifier including organoid volume and circularity. (*n* = at least 150 organoids from 3 biological replicates). Error bars: mean ± SD; ***, *p* < 0.001, *, *p* < 0.05. Wilcoxon rank-sum test and Mann-Whitney test (**G**,**H**). See also Figure S1.

Next, we investigated how hydrogel composition affects the morphology of mature PDAC93 organoids. Three predominant morphologies were identified: cystic, characterized by a central lumen surrounded by a monolayer of cells; solid, consisting of a compact, convex cluster of cells; and invasive, which includes a variety of compact morphologies displaying elongated extensions that penetrate the surrounding hydrogel (Figure 3A,B). Machine-learning-based morphological classification revealed that most organoids cultured in Matrigel display cystic morphology (58%), followed by solid (34%) and a small proportion displaying invasive features (8%) (Figure 3C). Conversely, collagen-containing hydrogels promoted the presence of invasive morphologies (53% CM, 46% C) along with moderate representation of solid (29% CM, 33% C) and fewer cystic (18% CM, 21% C) morphologies (Figure 3C). TGFβ conditioning had a marked effect on organoid morphology. In Matrigel, TGFβ led to a dramatic increase in solid forms (91%), while in CM and C hydrogels it shifted the morphology distribution further toward invasive phenotypes (63% CM-TFGβ, 54% C-TFGβ) (Figure 3C).

This morphological heterogeneity was further illustrated by t-SNE plots, which grouped organoids into distinct clusters based on morphology in both untreated (Figure 3D) and TGFβ-treated organoids (Figure 3E). A more nuanced view of morphological distribution by hydrogel type and treatment condition is provided in Figure (Figure S1A,B). Finally, when examining organoid morphology regardless of hydrogel or media conditioning (Figure 3F), the three main morphological categories remained clearly separable. The most discriminative descriptors used by our classifier (Table S3) were circularity, convexity, sphericity, geodesic elongation, and lumen radius. Cystic forms were consistently larger than both solid and invasive types in non-TGFβ-treated hydrogels and were also significantly larger than any organoids found in TGFβ-treated conditions (Figure 3G). As expected, invasive phenotypes exhibited lower circularity compared to solid or cystic organoids, regardless of hydrogel type (Figure 3H). Moreover, organoids cultured in Matrigel, both cystic and solid, displayed significantly higher circularity than those in CM and C hydrogels. This trend was also observed for convexity and sphericity, and inversely for geodesic elongation (Figure S1C–E). Finally, lumen radius positively correlated with organoid volume across conditions (Figure S1F).

### 2.4. Clinical Subtype and EMT Status of PDAC93 Organoids Are Influenced by ECM Composition and TGFβ

**Figure 4:**
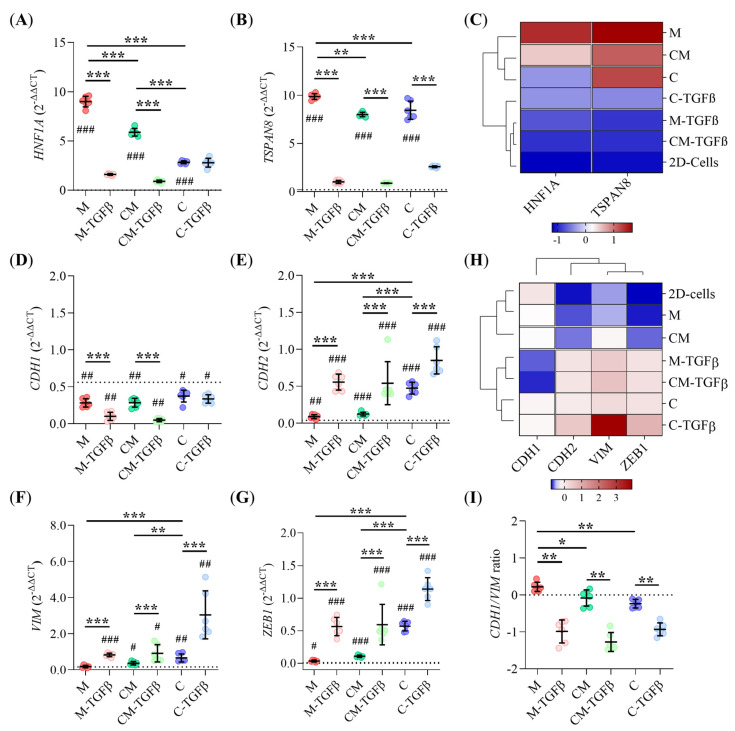
ECM stiffening and TGFβ drive mature PDAC93 organoids toward a basal-like state and upregulate EMT-related genes. (**A**,**B**) Relative mRNA expression levels of HNF1A and TSPAN8, respectively, in PDAC93 organoids cultured in M, CM, and C hydrogels, under regular media or TFGβ conditioning. The dotted line represents mRNA expression levels in 2D-plated cells. (*n* = 6 biological replicates); (**C**) Hierarchical heat-map showing the expression (Z-score) of clinical subtype markers in PDAC93 organoids, as extracted from panels A and B; (**D**–**G**) Relative mRNA expression levels of CDH1, CDH2, VIM, and ZEB1, respectively, in mature PDAC93 organoids cultured in M, CM, and C hydrogels, under regular media or TFGβ conditioning. The dotted line represents mRNA expression levels in 2D-plated cells. (*n* = 6 biological replicates); (**H**) Hierarchical heat-map showing the expression (Z-score) of EMT markers in PDAC93 organoids, as extracted from panels A to D; (**I**), Quantification of CDH1/VIM ratio. The dotted line represents the threshold distinguishing epithelial from mesenchymal status. Error bars: mean ± SD; *** or ^###^, *p* < 0.001, ** or ^##^, *p* < 0.01, * or ^#^, *p* < 0.05. One-way ANOVA and Paired *t* test (**A**,**B**,**D**,**F**). Wilcoxon rank-sum test and Mann-Whitney test (**E**–**G**). Two-way MANOVA (**D**–**G**). (#) Refer to the statistical differences between each hydrogel type and the2D-plated group. See also Figure S2.

To assess whether PDAC93 organoids grown in engineered hydrogels recapitulate the phenotypic plasticity observed in clinical samples, we first analyzed the expression of HNF1A and TSPAN8, two canonical markers of the classical PDAC subtype. As shown in Figure 4A,B, PDAC93 organoids cultured in Matrigel exhibited the highest expression of both markers. In contrast, expression levels were markedly reduced in PDAC93 organoids grown in CM and C hydrogels, correlating with the increasing collagen-I content, respectively. As expected, TGFβ conditioning strongly suppressed the expression of both genes across all hydrogel compositions. This trend is further reflected in the hierarchical heatmap (Figure 4C), where TGFβ-treated PDAC93 organoids cluster together, displaying low-expression levels of classical markers, comparable to those observed in 2D cultures. These data suggest that increased collagen-I content, particularly in C hydrogels, promotes a transcriptional shift from a classical toward a basal-like subtype, a transition further reinforced by TGFβ signaling. Consistent with these findings, hierarchical clustering of the main scaffolding proteins in each hydrogel revealed that collagen-containing hydrogels (C and CM) resemble an activated stroma profile (Figure S2), as described by Moffitt et al. [7], whereas Matrigel clustered with a normal stroma profile, underscoring the pivotal role of ECM composition in shaping PDAC subtype identity.

Next, we explored whether ECM composition modulates the transcriptional state of a custom EMT gene signature: E-cadherin (CDH1), N-cadherin (CDH2), vimentin (VIM), and ZEB1 [35]. A two-way MANOVA revealed significant main effects on RNA expression driven by both hydrogel composition (ηp^2^ = 0.623, *p* < 0.001) and the presence of TGFβ (ηp^2^ = 0.883, *p* < 0.001). Additionally, a significant yet moderate interaction was observed between both factors (ηp^2^ = 0.383, *p* < 0.001). Follow-up univariate ANOVA identified CDH1 (ηp^2^ = 0.717) and ZEB1 (ηp^2^ = 0.747) as the genes more influenced by hydrogel composition, with hydrogel C exerting the strongest effect on gene expression. Regarding media conditioning, ZEB1 (ηp^2^ = 0.768) and CDH1 (ηp^2^ = 0.672) are also the most affected genes, with TGFβ exerting a stronger impact on the EMT signature compared to the regular media.

Tukey’s corrected post-hoc analysis confirmed the down-regulation of the epithelial gene CDH1 and concurrent over-expression of mesenchymal markers (CDH2, VIM, and ZEB1) between 2D and 3D cultures, particularly within the C hydrogel or under TGFβ conditioning (Figure 4D–G). Among 3D hydrogels, no significant differences were observed in CDH1 expression (Figure 4D) while CDH2, VIM and ZEB1 expression levels were significantly up-regulated in C hydrogels compared to M or CM (Figure 4E–G). The presence of TGFβ further enhanced the expression of all genes analyzed. These findings are summarized in the hierarchical heat-map (Figure 4H), which shows two primary gene clusters: one centered on CDH1, and the other on CDH2/VIM/ZEB1. This pattern illustrates how the downregulation of CDH1 and the up-regulation of CDH2/VIM/ZEB1 underlie the EMT transition. Notably, the EMT signature of Matrigel-cultured organoids closely resembles that of the 2D cultures, while CM organoids have a transitional phenotype between M and C organoids, which exhibit marked EMT reprogramming. Moreover, TGFβ conditioning uniformly drove organoids toward a mesenchymal-like state, with the exception of C hydrogels, where the EMT profile was already established by the ECM itself. Finally, the CDH1/VIM ratio [36], a recognized EMT metric, confirmed that organoids in CM and C hydrogels, as well as all TGFβ-treated organoids, adopted a predominantly mesenchymal phenotype (CDH1/VIM ratio < 0), while those grown in M hydrogels retained a predominantly epithelial identity (CDH1/VIM ratio > 0) (Figure 4I).

### 2.5. Hydrogel Composition and TGFβ Alter the Subcellular Localization of EMT Markers in PDAC93 Organoids

**Figure 5:**
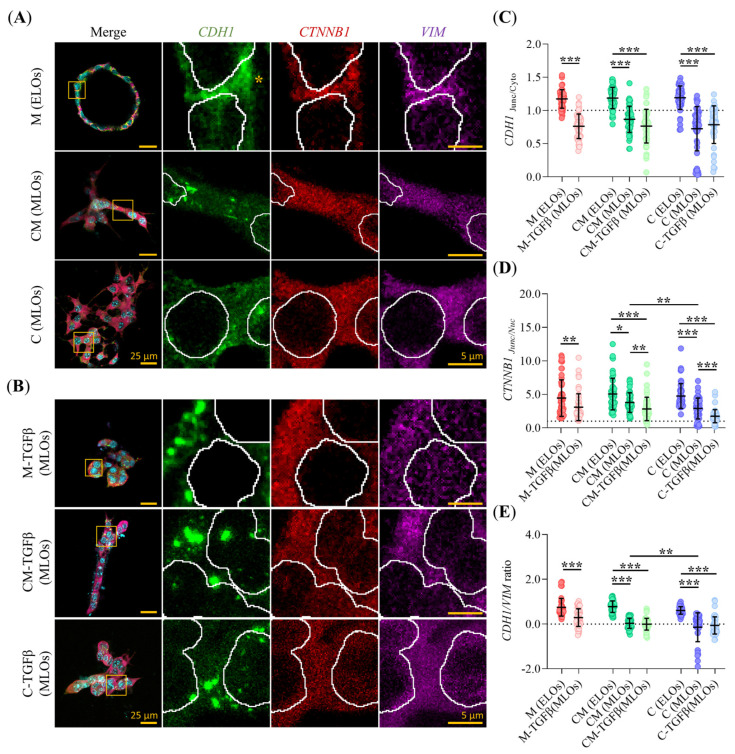
Analysis of the spatial distribution of EMT markers. (**A**,**B**) Representative Z-stack projections of CLSM images in the different mature PDAC93 organoid classes grown within M, CM and C hydrogels under regular or TFGβ conditioning, respectively. Staining: E-cadherin (CDH1, green), β-catenin (CTNNB1, red), vimentin (VIM, magenta), and nuclei (SiRDNA, cyan). Scale bars: 25 µm for low-magnification images (**left panel**) and 5 µm for high-magnification images (**right panel**). Yellow square insets show representative cell-to-cell junctions. Yellow asterisks indicate the location of CDH1 at the cell-cell junctions; (**C**,**D**) Quantification of EMT marker distribution extracted from the images in panel A and B, including: the CDH1 Junctional/Cytoplasm ratio and the CTNNB1 Junctional/Nuclei ratio, respectively. The dotted line indicates a ratio of 1. (*n* = 50 images from 3 biological replicates); (**E**) Quantification of the CDH1/VIM ratio. The dotted line represents the threshold distinguishing epithelial from mesenchymal status. (*n* = 50 images from 3 biological replicates). Error bars: mean ± SD; ***, *p* < 0.001, **, *p* < 0.01, *, *p* < 0.05. Wilcoxon rank-sum test and Mann-Whitney test (**C**–**E**).

We next analyzed whether hydrogel composition and TGFβ conditioning affect the subcellular localization of key EMT markers, CDH1, VIM and β-catenin (CTNNB1) as a function of hydrogel type and organoid morphology. Based on the phenotypic features and EMT profiles described in previous sections, we reclassified cystic organoids as epithelial-like organoids (ELOs), and grouped solid and invasive organoids under the category mesenchymal-like organoids (MLOs) to streamline comparative analysis. Representative confocal images of these EMT markers in ELOs and MLOs across the different hydrogel conditions, with or without TGFTGFβ, are shown in Figure 5A,B. Quantitative analysis of the junction-to-cytoplasm ratio of CDH1 revealed a consistent shift toward cytoplasmic localization in MLOs compared to ELOs, regardless of hydrogel composition or media conditioning (Figure 5C). In line with these findings, ELOs displayed continuous CDH1 staining at cell-cell junctions, while MLOs exhibit a patchy distribution of CDH1 in the cytoplasm (Figure 5A,B). Similarly, the junction-to-nucleus ratio of CTNNB1 followed the same trend observed for CDH1, with MLOs exhibiting prominent nuclear translocation of CTNNB1 compared to ELOs, regardless of hydrogel composition or media conditions (Figure 5D). Although this shift occurred in all MLOs, it was significantly more pronounced in C-MLOs than in CM-MLOs. Additionally, TGFβ conditioning further enhanced CTNNB1 nuclear localization in MLOs relative to their non-conditioned counterparts. To complement these findings, we calculated the CDH1/VIM ratio (Figure 5E) as a molecular index of epithelial versus mesenchymal state. In all MLOs, including CM-MLOs and C-MLOs, this ratio was ≤0, consistent with a mesenchymal phenotype. Conversely, all ELOs, regardless of hydrogel or media conditioning, maintained a CDH1/VIM ratio > 0, supporting their epithelial identity. Interestingly, TGFβ conditioning did not shift the CDH1/VIM ratio in Matrigel-grown organoids (CDH1/VIM ratio > 0).

### 2.6. Invasive PDAC93 Organoid Morphologies Trigger Extensive Collagen-I Remodeling

**Figure 6:**
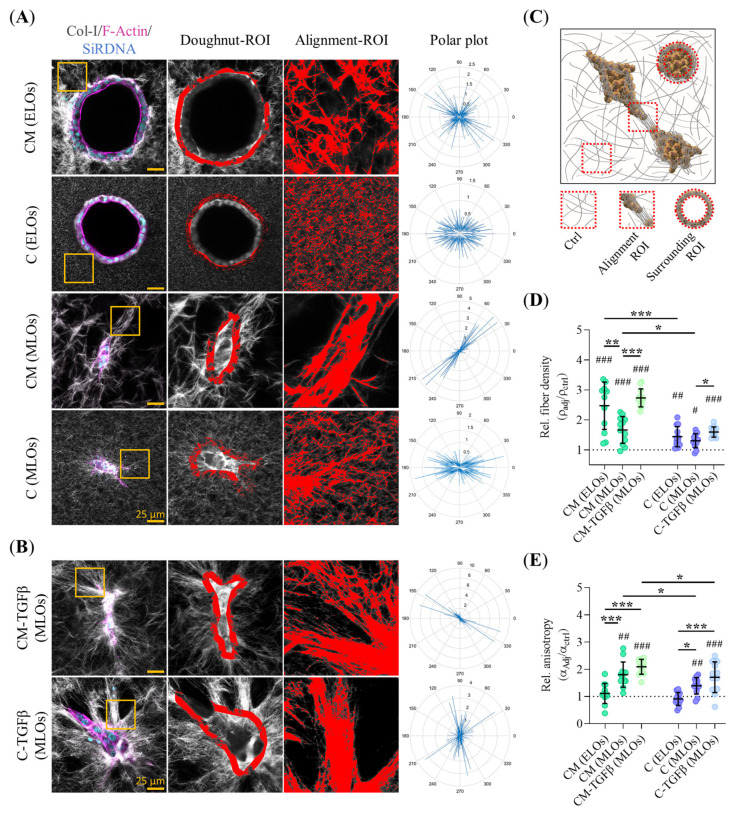
Invasive mature PDAC93 organoid morphologies correlate with higher levels of collagen-I remodeling. (**A**,**B**) Representative Z-stack projections of 3D SGH MP-CLSM images of collagen-I remodeling in the different PDAC93 organoid classes grown within CM and C hydrogels under regular or TFGβ conditioning, respectively. Staining: F-Actin (magenta) and nuclei (SiRDNA, cyan). The doughnut ROI illustrates fiber segmentation surrounding the organoids (7-microns band). Yellow square insets show representative areas with fiber remodeling. The alignment ROI and the polar plots highlight fiber alignment extracted from the yellow square inset. Scale bars: 25 µm; (**C**) Schematic representation of organoids (orange), fibers (gray), and the control, alignment, and surrounding ROIs used for quantification; (**D**,**E**) Quantification fiber compaction and alignment (anisotropy, α), respectively, extracted from panel A and B. The dotted line represents fiber remodeling in the control ROI. (*n* = 12 images from 3 biological replicates). Error bars: mean ± SD; *** or ^###^, *p* < 0.001, ** or ^##^, *p* < 0.01, * or ^#^, *p* < 0.05. One-way ANOVA and Paired *t* test (**D**,**E**). (#) Refer to the statistical differences between each hydrogel type and the 2D-plated group.

We analyzed collagen-I fiber densification and alignment around mature PDAC93 organoids by SHG imaging. Representative images of the collagen-I mesh and a schematic of the image-analysis workflow are shown in Figure 6A–C. Quantitative analysis revealed that both ELOs and MLOs, either conditioned with TGFβ or not, induced local collagen I densification in all hydrogels (Figure 6D). However, the degree of densification was significantly higher around organoids grown in mixed-composition hydrogels (CM-ELOs and CM-MLOs) compared to those grown in collagen-only (C-ELOs and C-MLOs). Within CM hydrogels, CM-ELOs generated higher densification compared to their mesenchymal-like CM-MLO counterparts. We also assessed fiber alignment via the anisotropy index (α). MLOs, but not ELOs, exhibited significant collagen-I alignment surrounding the organoid boundary, regardless of the hydrogel (Figure 6E). Among MLOs, CM-MLOs produced enhanced collagen-I fiber alignment compared to C-MLOs. Finally, TGFβ conditioning further increased collagen-I fiber alignment in PDAC93 organoids within CM hydrogels.

### 2.7. ECM Stiffening Rewires Metabolic Activity in PDAC93 Organoids

**Figure 7:**
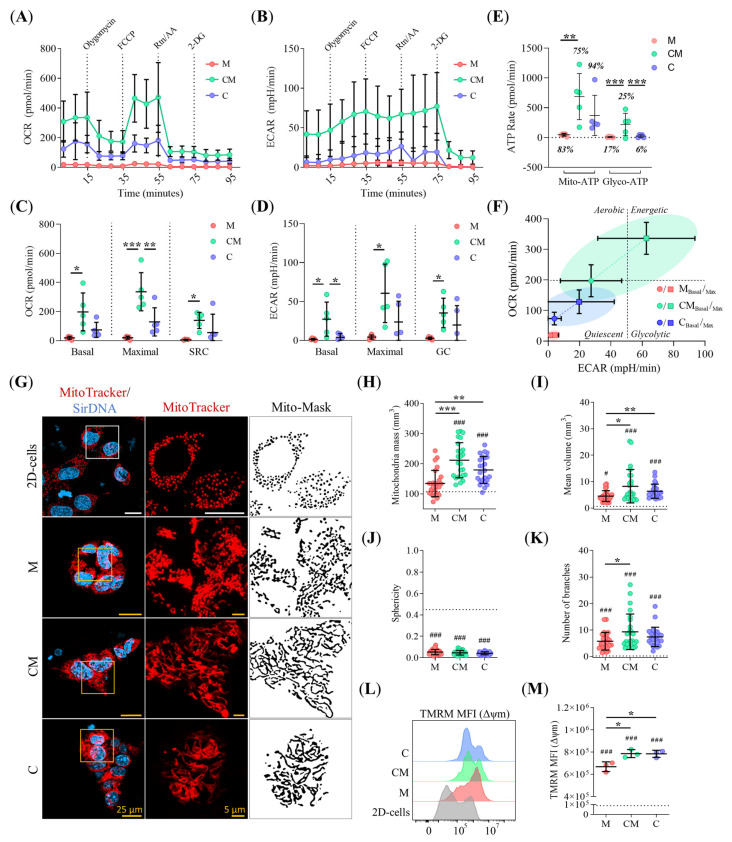
Collagen containing hydrogels promote highly bioenergetic mature PDAC93 organoids. (**A**,**B**) Representative OCR (pmol/min) and ECAR (mpH/min) curves over time in PDAC93 organoids grown within M, CM, and C hydrogels; (**C**) Basal and maximal respiration rates, as well as SRC (pmol/min), extracted from panel A. (*n* = 5 biological replicates); (**D**) Basal and maximal proton flux, as well as GC rate (mpH/min), extracted from panel B. (*n* = 5 biological replicates); (**E**) ATP production rate (pmol/min), showing contributions (%) from mito-ATP and glyco-ATP relative to total ATP production. (*n* = 5 biological replicates); (**F**) Energetic map depicts basal (circle) and maximal (square) OCR and ECAR levels in PDAC93 organoids grown in M, CM, and C hydrogels. The four quadrants represent metabolic states: “Quiescent,” “Aerobic,” “Energetic,” or “Glycolytic.” Colored ellipses illustrate the metabolic space occupied by organoids within each hydrogel condition; (**G**) Representative Z-stack projections of CLSM images of PDAC93 organoids grown within CM and C hydrogels, alongside 2D-plated cells, under standard media conditions. Staining: mitochondria (MitoTracker, red) and nuclei (SiRDNA, cyan). Yellow square insets highlight mitochondrial morphology. Scale bars: 25 µm for low-magnification images (left panel) and 5 µm for high-magnification images (right panel); (**H**–**K**) Quantification of mitochondrial morphological descriptors including: mitochondrial mass (mm^3^), mean volume (mm^3^), sphericity, and number of branches, respectively, extracted from panel G. The dotted line indicates levels in 2D-plated cells. (*n* = at least 300 cells from 3 biological replicates); (**L**–**M**) Representative FACS histograms and MFI values for TMRM staining (Δψm) in PDAC93 organoids grown within M, CM, and C hydrogels, alongside 2D-plated cells. The dotted line indicates levels in 2D-plated cells. (*n* = 3 biological replicates). Error bars: mean ± SD; *** or ^###^, *p* < 0.001, **, *p* < 0.01, * or ^#^, *p* < 0.05. One-way ANOVA (**C**–**E**,**J**,**M**). Wilcoxon rank-sum test (**H**,**I**,**K**). (#) Refer to the statistical differences between each hydrogel type and the 2D-plated group.

To determine how ECM biomechanical properties influence cellular metabolism, we measured oxygen consumption rate (OCR) and extracellular acidification rate (ECAR) in PDAC93 organoids cultured in M, CM, and C hydrogels. Organoids grown in CM hydrogels displayed significantly elevated basal and maximal respiration rates, along with enhanced SRC, compared to those in M and C hydrogels (Figure 7A,C). Moreover, ECAR measurements further revealed that CM-grown organoids showed glycolytic activity across basal, maximal, and GC rates compared to those in M hydrogels (Figure 7B,D). Although overall ECAR values were comparable between CM and C conditions, CM organoids showed significantly higher basal glycolysis. (Figure 7B,D). ATP production profiling indicated that PDAC93 organoids predominantly rely on oxidative phosphorylation (OXPHOS) for energy, regardless of the ECM composition (Figure 7E). However, our results confirmed that glycolysis also contributed to the energy pool, particularly in CM-grown organoids, which exhibited significantly elevated Glyco-ATP production relative to M and C (Figure 7E). Consequently, the metabolic profile of the PDAC93 organoids, defined by their basal OCR-ECAR ratio, revealed short energetic demands for M and C grown organoids, and a high energy-consuming phenotype for CM grown organoids (Figure 7F).

To gain mechanistic insights into these metabolic differences, we analyzed mitochondrial morphology and membrane potential (Δψm) via CLSM imaging (Figure 7G). Our analysis revealed significant increased mitochondrial mass in organoids cultured in CM and C hydrogels compared to M grown organoids (Figure 7H). In addition, mitochondria in CM and C exhibited enlarged and hyperfused morphologies, based on the level of branches, in comparison to M grown organoids (Figure 7I,K). No significant differences in mitochondrial sphericity were found between the hydrogels (Figure 7J). Enhanced Δψm levels were also detected in CM- and C-grown organoids (Figure 7L,M) compared to M grown organoids. In contrast, 2D cultures exhibited markedly lower mitochondrial mass, mean volume, fusion level, and Δψm than PDAC93 organoids (Figure 7H–M), regardless of the hydrogel composition, while promoting increased mitochondrial sphericity—a hallmark of dysfunctional mitochondria (Figure 7J). These findings suggest that 3D environments boost mitochondrial mass and metabolic rewiring, especially within CM hydrogels. More importantly, CM hydrogels promote a metabolically active phenotype characterized by simultaneous engagement of OXPHOS and aerobic glycolysis, consistent with the Warburg effect.

### 2.8. 3D-Environment Confers Adaptive GEM Resistance in PDAC93 Organoids in a Collagen-Dependent Manner

**Figure 8:**
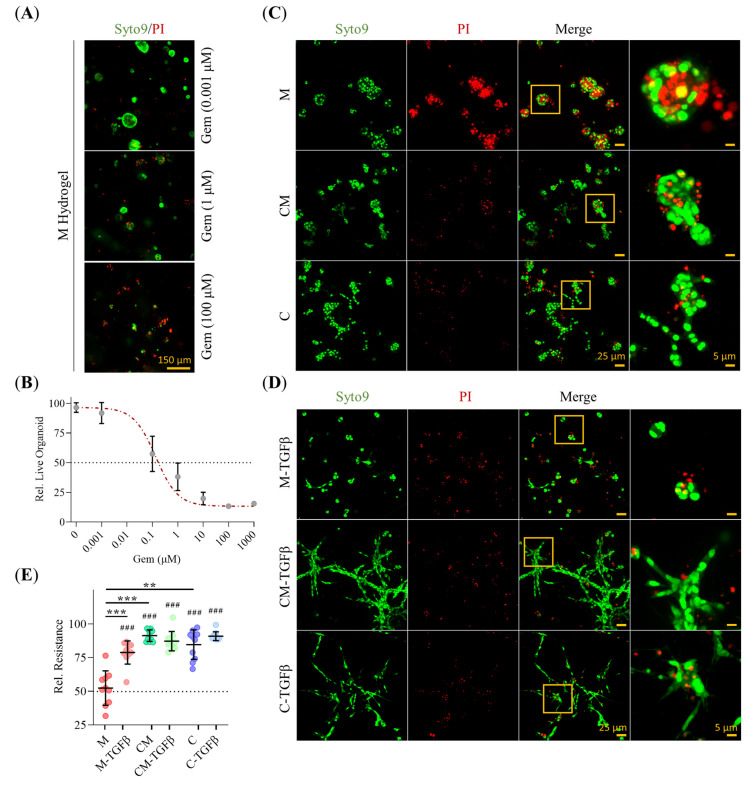
(**A**) Representative Z-stack projections of CLSM images of mature PDAC93 grown within M hydrogels treated with increasing concentrations of Gem (0.001, 1, and 100 μM). Staining: live nuclei (Syto9, green) and necrotic nuclei (PI, red). Scale bars: 150 µm; (**B**) IG_50_ dose-response curve extracted from the images in panel A. The dotted line indicates IG_50_ value. (*n* = 3 biological replicates); (**C**,**D**) Representative Z-stack projections of CLSM images of mature PDAC93 grown within M, CM, and C hydrogels, under regular or TFGβ conditioning, respectively, in the presence of 0.1 µM Gem. Staining: live nuclei (Syto9, green) and necrotic nuclei (PI, red). Yellow square insets highlight Gem-induced cytotoxicity. Scale bars: 25 µm for low-magnification images (left panel) and 5 µm for high-magnification images (right panel); (**E**) Quantification of organoid chemoresistance to Gem under regular or TFGβ conditioning, respectively. The dotted line represents sensitivity levels in 2D-plated cells. (*n* = at least 100 organoids from 3 biological replicates). Error bars: mean ± SD; *** or ^###^, *p* < 0.001, **, *p* < 0.01. Wilcoxon rank-sum test and Mann-Whitney test (**E**). (#) Refer to the statistical differences between each hydrogel type and the 2D-plated group.

To explore how ECM composition modulates drug resistance, we assessed PDAC93 organoid sensitivity to gemcitabine (Gem) using in vitro live/dead assays in 3D hydrogels. Prior to this, we established the IG_50_ dose-response curve using M hydrogel, selected due to their epithelial-like phenotype and presumed highest chemosensitivity (Figure 8A,B). Representative images of the Gem-treated organoids are shown in Figure 8C,D. Quantitative analysis revealed that PDAC93 organoids cultured within CM and C hydrogels exhibited approximately 40% higher resistance to Gem compared to those in M hydrogels (Figure 8E). Notably, no significant differences in drug response between CM and C organoids, regardless of the media used. TGFβ conditioning further enhanced Gem resistance in M organoids, bringing their survival to levels comparable to those grown in CM and C hydrogels. Intriguingly, 2D cultures exhibited similar sensitivity to Gem as M-grown organoids, suggesting that epithelial-like traits, rather than dimensionality alone, govern drug responsiveness.

### 2.9. ECM Composition Drives Clinical Subtype in PDAC93 Organoid-Derived Murine Model, Enhancing Tumor Aggressiveness and GEM-Resistance

**Figure 9:**
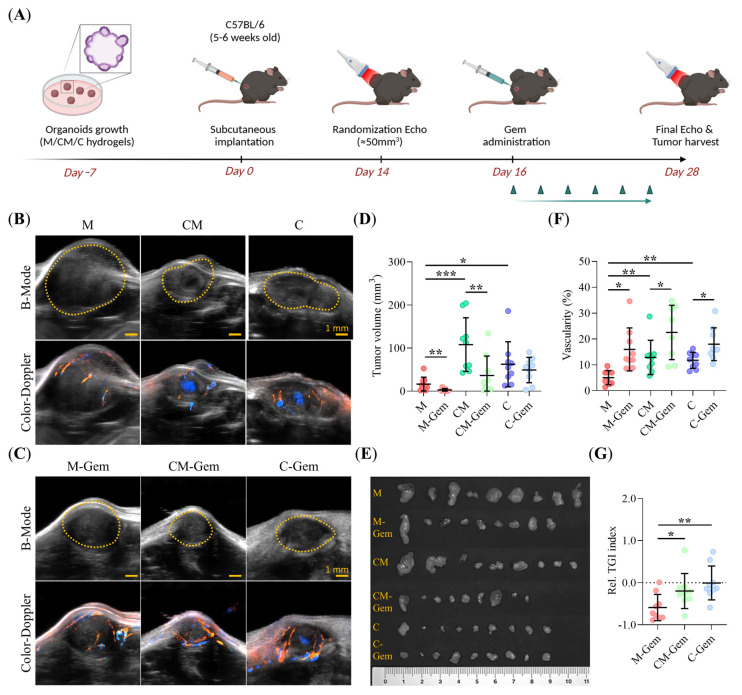
Collagen-enriched environment promotes tumor aggressiveness and impairs Gem efficacy in PDAC93 organoid-derived tumors. (**A**) Schematic of the experimental design. Gem (100 mg/kg) was administered intraperitoneal on days 16, 18, 20, 22, 24, and 26 (green triangles); (**B**,**C**) Representative 3D-rendered views of tumors 28 days after organoid injection, comparing groups with and without Gem treatment. B-mode images show anatomical structure along with surrounding and intra-tumoral vasculature (Color Doppler-mode, red and blue). The dotted yellow line indicates the tumor margins. Scale bars: 1 mm; (**D**,**E**) Quantitative analysis of tumor volume and vasculature, respectively, extracted from panel B and C. (*n* = 9); (**F**) Photographs of excised tumors harvested on day 28; (**G**) Quantification of the relative tumor growth inhibition index (TGI) in the Gem-treated groups. The dotted line indicates the threshold separating tumor regression (TGI < 0) and tumor progression (TGI > 0). (*n* = 9); Error bars: mean ± SD; ***, *p* < 0.001, **, *p* < 0.01, *, *p* < 0.05. Wilcoxon rank-sum test and Mann-Whitney test (**D**,**F**). One-way ANOVA (**G**). (See also Figure S3).

To validate our in vitro findings in a preclinical context, we assessed how ECM composition affects tumorigenic potential, vascularization, and chemotherapeutic response in vivo. To this end, PDAC93 organoids previously cultured in M, CM, and C hydrogels were subcutaneously implanted into immunocompetent, syngeneic C57Bl/6 mice (*n* = 9/group). Gem treatment was initiated on day 16, once tumors reached a volume of 50 mm^3^, and tumor growth was monitored by echography until day 28, prior to sacrifice (Figure 9A). At this time, PDAC93 tumors derived from organoids cultured in CM and C hydrogels displayed significantly larger normalized volumes compared to those derived from M hydrogels (Figure 9B–D). In contrast, Gem treatment led to a marked reduction in tumor volume for organoid-derived tumors grown in both M and CM hydrogels (~80% and ~70% reduction, respectively) compared to untreated controls. however, C-derived tumors were notably resistant to Gem, as evidenced by no significant difference in tumor size between the C and C-Gem groups (Figure 9C,D). These trends were corroborated by tumor images collected on day 28 (Figure 9E). Histological analysis confirmed the clinical subtype plasticity associated with the presence of collagen-I observed in vitro. Indeed, we observed statistically significant down-regulation of the classical GATA6 marker with increasing collagen-I levels (Figure S3). Furthermore, tumors derived from CM and C hydrogels displayed increased peri- and intra-tumoral vasculature compared to M-derived tumors (Figure 9B–F), while Gem treatment enhanced tumor vascular density across all hydrogel types (Figure 9C–F). Quantification of treatment efficacy using the Tumor Growth Inhibition (TGI) index demonstrated markedly higher Gem sensitivity in Gem-treated tumors derived from M organoids compared to those grown in CM and C hydrogels (Figure 9G). Notably, CM-Gem and C-Gem groups exhibited similar TGI, with C-derived tumors displaying particular chemoresistance to Gem. Overall, these results suggest that the presence of Collagen-I boosts tumor chemoresistance in our murine model.

## 3. Discussion

This study provides a comprehensive analysis of the impact of the composition and biomechanical properties of the scaffolding material on the phenotype, metabolism and drug resistance of murine PDAC93 organoids. PDAC93 organoids were cultured in three different hydrogels: Matrigel-only (M), representing a biochemically rich yet mechanically soft environment; collagen-only (C), characterized by a highly structured and rigid bio-functional environment with limited biochemical complexity; and a collagen-Matrigel (CM) mixture, which combines robust structural scaffolding with biochemical richness. To assess their impact on organoid behavior, the mechanical properties of each hydrogel were first characterized. C hydrogels exhibit higher rigidity, elastic and viscous components than M and CM hydrogels. This mechanical environment is known to enhance cell migration by enabling cells to rapidly generate mechanical tensions [37]. Consistently, our EOSs embedded in C hydrogels exhibited the highest invasive capacity among all three hydrogels tested. This enhanced 3D migration in rigid hydrogels, driven by collagen-I concentration, has been reported to promote collective cell migration and mechanical stability, facilitating organoid development in various cancer types, including liver [38], colon [39], breast [40], or brain [41]. In CM hydrogels, the increased concentration of Matrigel results in a reduction of both the rigidity and viscosity of the hydrogel [42]. This explains the lower EOSs migration dynamics seen in CM hydrogels compared to C hydrogels. However, this reduction less pronounced than anticipated, which can be explained by the larger CM pore size, as well as by mechanobiological changes elicited by Matrigel. Indeed, using mixed CM hydrogels, we have previously demonstrated [25] that low concentrations of Matrigel enhance mesenchymal cancer cell migration by stimulating β1 integrin expression and the formation of small focal adhesions, which in turn enhance cell tractions. However, an excess of Matrigel leads to the formation of large focal adhesions, which favor attachment over traction and ultimately reduce migration rates [25,43]. TGFβ stimulation further increases EOSs migration speed and protrusive activity across all hydrogel types, with a more pronounced effect in collagen-containing matrices. We also evaluated the morphology of mature organoids grown within our hydrogels. Organoids cultured in M, where EOSs exhibit a static behavior, predominantly display large cystic morphologies, whereas highly motile EOSs in CM and C hydrogels mostly develop into smaller solid or invasive organoids. Notably, the addition of TGFβ promotes the formation of fewer, small, solid and invasive organoids across all hydrogel types, irrespective of their composition. This suggests that the EOSs migration dynamics correlate with the maturation of organoids into epithelial cystic forms (slow EOSs), or into solid or invasive mesenchymal-like structures (fast EOSs). TGFβ however overrides hydrogel-specific effects, by facilitating a pro-migratory EOSs phenotype that drives organoid morphologies exclusively towards solid and invasive mesenchymal-like morphologies.

The heterogeneity of PDAC tumors poses a significant challenge for clinical stratification [44]. Both our analysis of the expression of clinical subtype markers HNF1A and TSPAN8 [45], and the characterization of the hydrogel composition point in the same direction: collagen-I containing hydrogels (CM and C), recapitulate features of an “activated” stroma [7], where PDAC93 organoids display a clinical phenotype that transitions from classical to basal with increasing concentrations of collagen-I. Conversely, PDAC93 organoids grown in Matrigel-only (M) hydrogels, representative of a “normal” stroma [7], display a predominantly classical clinical phenotype. These findings are also supported by our in vivo results, as the subcutaneous tumors exhibited a trend toward the classical marker GATA6 down-regulation [46] with increasing concentration of collagen-I. In summary, our results reflect how the composition and properties of the ECM modulate the clinical subtype of the PDAC93 organoids grown from basal PDAC93 cells, giving rise to a more classical or basal phenotype as a function of Matrigel and collagen-I content. This is consistent with our morphological analyses, as PDAC93 organoids do not exhibit a uniform morphology, across different hydrogel compositions, and it is the increasing concentration of collagen-I that drives the transition from a higher frequency of epithelial-like cystic forms to more intermediate solid and invasive forms.

EMT involves the replacement of epithelial cytokeratin with mesenchymal vimentin and is associated with the depletion of CDH1 and the activation of CDH2 at cell junctions [47]. Our qRT-PCR analysis confirmed significant changes in the expression of CDH2, ZEB1 and VIM in collagen-I containing hydrogels. Interestingly, CDH1 expression was similarly downregulated in all M, CM and C hydrogels, in contrast with recent studies that link PDAC ECM rigidity to CDH1 suppression and poor clinical outcomes [26,48,49]. However, our immunofluorescence analysis revealed CDH1 delocalization from the membrane to the cytoplasm, particularly in MLOs, supporting findings by Aiello et al. [50], who explained CDH1 loss in PDAC tumors as membrane delocalization rather than transcriptional down-regulation. Similarly, it has been recently reported that pancreatic carcinomas retain CDH1 expression, despite exhibiting high invasiveness and metastatic potential [49]. We also observed CTNNB1 delocalization from the membrane to the cytoplasm or nucleus, particularly in MLOs. Although complete CTNNB1 nuclear translocation was not observed, cytoplasmic accumulation of CTNNB1, a well-established hallmark of EMT across various cancer types [51,52], was evident in all 3D hydrogels, but especially in MLOs. These findings align with observations by Zhang et al. [53], who reported similar CTNNB1 patterns in PanIN lesions in KPC mice. In summary, PDAC93 organoids grown in C hydrogels lean towards a pure EMT mesenchymal phenotype, those grown in mixed CM hydrogels display an intermediate phenotype, and M hydrogels preserve a mostly epithelial phenotype, phenotypically similar to 2D cultures. These phenotypic differences are further supported by CDH1/VIM expression ratios, assessed by both qRT-PCR and immunofluorescence. As expected, TGFβ-conditioned hydrogels consistently induce a pure mesenchymal phenotype, marked by strong ZEB1 and VIM over-expression and CDH1 downregulation [54]. Both CDH1 and CTNNB1 were also delocalized from the membrane under these conditions, underscoring the role of TGFβ in EMT in PDAC93 tumors. These results are consistent with the previously described modulatory role of collagen-I in shaping PDAC93 organoid morphology and clinical subtype.

As EMT promotes ECM remodeling, we analyzed whether our organoids also replicated that behavior. Our analysis showed collagen-I densification around organoids across all hydrogel types, with the strongest effects in collagen-Matrigel mixed hydrogels (CM-ELOs and CM-MLOs). Collagen-I alignment, however, occurred exclusively in MLOs, with the most pronounced effect in CM hydrogels and under TGFβ conditioning. These findings align with reports that identified ZEB1 upregulation as a driver of collagen-I remodeling through LOX-mediated activation [55]. Indeed, in PDAC tumors, stromal populations extensively accumulate fibrous ECM components, promoting EMT and ECM remodeling [56]. This heterogeneous deposition and linearization of fibers in the TME promote cell invasiveness, as confirmed by EOSs migration in our collagen-based hydrogels [57]. In summary, our results suggest that hydrogels of mixed-composition (CM) best mimic the desmoplastic ECM of PDAC tumors. This supports recent studies reporting that ECM stiffening and/or remodeling activate latent TGFβ signaling, fostering tumor invasiveness, drug resistance, and immune evasion [58].

As EMT induces metabolic rewiring [59], we analyzed glucose consumption in our PDAC93 organoids, to assess whether 3D arrangements elicited enhanced mitochondrial bioenergetics compared to 2D configurations [60], particularly in organoids cultured within hydrogels of mixed composition (CM), which show increased glycolytic activity, consistent with the Warburg effect, while simultaneously maintaining elevated OXPHOS levels. This metabolic duality supports PDAC cell dissemination and metastasis [61] as PDAC cells have been shown to reprogram their metabolism in response to matrix rigidity, amplifying the Warburg effect and tumor malignancy. Moreover, under confined stiff ECM conditions, PDAC cells have been reported to exhibit mitochondrial remodeling, including increased mitochondrial mass, fusion, and membrane potential [62]. Our data are consistent with these findings in 3D hydrogels, with CM and C hydrogels demonstrating the highest mitochondrial remodeling levels, a hallmark of enhanced metastatic potential in PDAC tumors [63]. Collectively, these findings suggest that CM hydrogels closely mimic the metabolic and phenotypic behaviors of PDAC tumors, which are characterized by upregulated, aberrant glycolytic flux, even under normoxia. These metabolic adaptations confer both invasive and chemoresistance advantages to PDAC cells, as observed in both patient-derived tissues and mouse models [64].

EMT has been shown to contribute to tumor aggressiveness and resistance [65,66,67,68] along with matrix stiffness [13] remodeling and metabolic rewiring [69]. Consequently, we complemented our study by performing an analysis of PDAC93 sensitivity to Gem. Consistent with recent findings, our in vitro results showed that the increased stiffness of collagen-based matrices correlates with higher chemoresistance to Gem compared to softer hydrogels like Matrigel or 2D cultures [70,71]. Likely, the increased fiber deposition and alignment observed in collagen-based hydrogels further exacerbate Gem resistance by restricting drug diffusion [72,73]. Notably, Gem-resistant PDAC93 cells exhibited higher expression of EMT markers (e.g., ZEB1) and reduced levels of CDH1 [35,74]. Indeed, EMT status, often quantified by the CDH1/VIM ratio, has been proposed as a prognostic factor for PDAC tumors’ response to Gem, correlating with increased organoid viability under TGFβ conditioning [36]. Similarly, highly bioenergetic profiles, including increased glycolytic flux and mitochondrial mass observed in CM- and C-based hydrogels, are also associated with Gem resistance in PDAC cells [75,76]. This is also supported by our results obtained using an immunocompetent mouse model, as syngeneic CM-derived subcutaneous tumors foster the highest rates of vascularization and tumor growth, confirming recent studies that highlight how ECM stiffness induces neovascularization, thereby creating a perivascular niche that facilitates cancer metastasis [77]. Interestingly, Gem administration increased tumor vascular density regardless of hydrogel composition and significantly reduced metabolic activity, ultimately resulting in slower tumor growth [78]. These results suggest that a dense, fibrotic ECM in the immediate cellular environment, primarily composed of collagen, impedes drug diffusion while promoting an aggressive, metabolically active phenotype, which contributes to heightened Gem chemoresistance. Notably, mixed-composition (CM) hydrogels closely replicate the PDAC TME by recreating biomechanical stimuli and bioactive cues that better support the delicate balance between tumor growth and vascular development.

## 4. Conclusions

**Figure 10:**
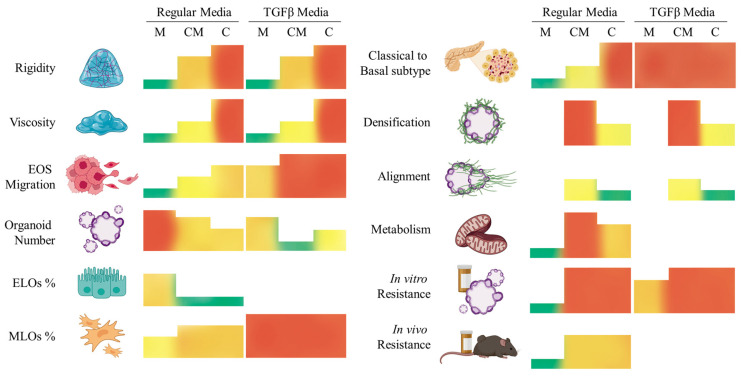
A visual summary of the quantitative results obtained from the primary experiments conducted in this study. The color scale ranges from green to red, indicating an ascending effect.

In summary, we have demonstrated that the composition and biomechanical properties of the 3D hydrogel modulate the behavior of PDAC93 tumor organoids. These properties are critical to create an environment that recapitulates the native tumor microenvironment. Our data further show that tuning these hydrogel properties enables the mimicry of the plasticity and heterogeneity of pancreatic cancers, which often contain both regions of classical and basal phenotypes. As illustrated in the summary Figure 10, we postulate that mixed hydrogels composed of collagen-I and Matrigel (CM) most accurately replicate what is found in the clinic. Indeed, CM hydrogels approximate an activated stroma where both epithelial and mesenchymal PDAC93 tumor organoids coexist and can be considered as existing in a transitional state between classical and basal phenotypes. Furthermore, this environment promotes the development of invasive, desmoplastic, metabolic, vascularized, and chemo resistant PDAC93 organoids that closely resemble PDAC tumors found in the clinic. Pure Matrigel (M) hydrogels, representing a normal stroma, give rise to organoids of a purely classical phenotype-. Finally, collagen-I only (C) hydrogels and any hydrogel conditioned with TBFβ give rise to organoids with an overly aggressive phenotype that can be considered purely of basal subtype. Therefore, they should only be used to simulate low-frequency purely basal tumors.

## 5. Materials and Methods

### 5.1. Micro-Device Design and Fabrication

3D organoids were grown in homemade polydimethylsiloxane (PDMS) microfluidic devices containing three 5 mm diameter hydrogel-loading wells enclosed in a 1.2 mL cylindrical media reservoir. The schematic rendering of the devices is shown in Figure S4A,B. A 3D CAD model (Figure S5A) of the master mold used to fabricate the micro-devices was generated using Inventor 2020 (Autodesk, San Francisco, CA, USA), and 3D-printed in a Form3 printer (FormLabs, Somerville, MA, USA) using Tough 2000 resin (FormLabs, RS-F2-TO20-01) with an axial resolution of 50 μm (Figure S5B). The fabricated master mold was washed with 2-propanol in a Form Wash (FormLabs, Somerville, MA, USA) device and post-cured in a Form Cure UV oven (FormLabs, Somerville, MA, USA) at 80 °C for 2 h. The cylindrical reservoirs were fabricated by casting a 10:1 base-to-curing agent ratio PDMS mixture onto the 3D printed master mold, followed by degassing for 25 min to remove air bubbles, and polymer curation at 70 °C for 2 h. Subsequently, the cured PDMS was removed from the master mould and three holes were pierced through the material using a 5 mm hole-puncher to create the wells where the hydrogel will be loaded. Then, the PDMS micro-device was plasma bonded to a glass coverslip (#1 thickness, 35 mm diameter; Menzel Glaser, Braunschweig, Germany) using a Zepto plasma cleaning system (Diener Electronic, Ebhausen, Germany) at 85 W power and 0.3–0.4 mBar pure O_2_ pressure for 1 min. Figure S4C shows one of the fabricated devices. Prior to any experiment, the devices were UV sterilized for 15 min and incubated with 0.1 mg/mL poly-D-lysine solution in PBS at 37 °C for 2 h to improve hydrogel attachment to the hydrogel loading wells. Finally, the wells were rinsed with sterile deionized water and dried under sterile conditions.

### 5.2. Hydrogel Fabrication

Three different hydrogels were prepared using varying concentrations of collagen-I (BD Biosciences, Franklin Lakes, NJ, USA, 354249) and Matrigel growth factor reduced (GFR) (Corning, USA, New York, NY, USA, 354230). We refer to them as: M (4 mg/mL Matrigel), CM (2 mg/mL collagen-I, 2 mg/mL Matrigel) and C (4 mg/mL collagen). To fabricate these hydrogels, a mixture of 10× PBS and sterile deionized water was first prepared, following the addition of collagen-I and/or Matrigel at the desired concentration (pH = 7). Collagen (C) and CM hydrogel gelation was performed in two steps: the hydrogels were first incubated at 25 °C for 30 min and then at 37 °C for 15 min. M hydrogel was gelated in a single incubation step at 37 °C for 15 min.

### 5.3. Mechanical Measurement of Stiffness

The mechanical properties of hydrogels were characterized using a stress-controlled DHR-10 rheometer (TA Instruments, New Castle, DE, USA) with a plate-cone geometry, as described by Valero et al. [79]. Briefly, 1.2 mL of the hydrogel prepolymer was placed on the lower plate of the rheometer. The gap between the upper and lower plates was initially set to 0.1 mm and maintained constant throughout the gelation process. Once gelation was complete, the gap was reduced to 0.052 mm. Time sweep experiments were then performed for at least 1 h, applying a sinusoidal strain amplitude of 0.3% (within the linear viscoelastic regime, LVR) at a frequency of 0.1 Hz. The storage (G′) and loss (G″) modules of the hydrogels were recorded over time.

### 5.4. Hydrogel Morphology Measurement

The 3D microstructural characterization of hydrogels was performed through automated analysis of Z-stack images acquired using a LSM-880 inverted multiphoton excitation confocal laser scanning microscope (MP-CLSM) (Zeiss, Jena, Germany) equipped with a 63× C-Apochromat objective (1.2 NA, W). The microscope’s Mai-Tai^®^ DeepSee™ T-Sapphire laser (Spectra-Physics, Milpitas, CA, USA) was tuned at 790 nm for the Collagen-I fiber network’s SHG imaging. The fiber signal was filtered through a 380–420 nm band-pass filter before being directed to the Zeiss BiG-2 GaAsP detection module. Z-stack images, capturing a total volume of 135 × 135 × 50 µm^3^, were acquired while maintaining the hydrogels in organoid culture media at 37 °C and 5% CO_2_ to replicate culture conditions. The Collagen-I network was analyzed using a custom Fiji v2.16.0 (Bethesda, MD, USA) script [80]. Network geometry parameters, including Collagen-I fiber thickness, density, and pore diameter, were assessed using a previously established pipeline from our group [25].

### 5.5. Cell Line Culture and PDAC93 Organoid Generation

Pancreatic Ductal Adenocarcinoma 93 cells (PDAC93) (mouse, male) were originally isolated from pancreatic tumors Kras+/LSL-G12D; Trp53flox/flox; Ptf1aCre/+ (KPC) mice on a C57Bl/6J strain background. These cells generate a spectrum of ductal lesions resembling low- and high-grade human pancreatic intraepithelial neoplasia (PanINs), which can further progress into primary and metastatic pancreatic ductal adenocarcinoma (PDA) [16]. PDAC93 cells were cultured in Advanced DMEM/F12 medium (Gibco, Barcelona, Spain, 10570083) supplemented with 10% FetalClone III (Cytiva, Barcelona, Spain, 10570083) and 1% Penicillin/Streptomycin (P/E) (Gibco, 15140122) at 37 °C in a 5% CO_2_ atmosphere. The medium was refreshed every 2–3 days, and cells were passaged at sub-confluence every 3 days using 0.25% Trypsin-EDTA solution (Merck, Darmstadt, Germany, T4049). When required, PDAC93 cells were pre-conditioned for 48 h in standard DMEM/F12 medium supplemented with 1 ng/mL TGFβ (R&D Systems, Minneapolis, MN, USA, 240-B), which was maintained throughout the culture period. A detailed description of the culture media is provided in Table S1.

For organoid generation, PDAC93 or PDAC93-GFP cells were embedded in non-gelated M, CM, or C hydrogels at a final concentration of 3 × 10^5^ cells/mL. A 20 μL aliquot of the cell-hydrogel mixture was added to each hydrogel-loading well in the micro-device and allowed to gelate, as described in the Section 5.2. Following gelation, either regular or TGFβ-supplemented medium was added, and the 3D culture was incubated at 37 °C and 5% CO_2_ as required. At this stage, the loaded devices contained early organoid seeds (EOSs), consisting of small clusters of organoid-generating cells. Experiments involving “mature organoids” were conducted after 7 days of incubation.

HEK293T embryonic kidney cells (human, female) were exclusively used for lentivirus packaging and cultured according to the manufacturer’s instructions. 

### 5.6. Generation of PDAC93-GFP Cell Lines Using Lentiviral Transduction

Stably GFP-expressing PDAC93 cells were generated through lentiviral transduction of the parental PDAC93 cells. Lentiviruses were produced using X-tremeGENE DNA Transfection Reagent (Roche, Basel, Switzerland, 6366244001) and a third-generation Lentiviral Packaging Mix (Merck, Darmstadt, Germany, SHP001), following the manufacturers’ protocols. In brief, 5 × 10^5^ HEK293T/17 packaging cells (ATCC, CRL-11268) were seeded into 6-well plates in complete DMEM/F12 medium. To each well, a transfection mixture containing 2 μg of the pSIN-GFP-Fg plasmid (Addgene plasmid #174307; RRID:Addgene-174307), 5 μL of Lentiviral Mix, and 9 μL of X-tremeGENE Reagent was added dropwise, followed by a 20-min incubation at room temperature (RT). After transfection, 100 μL of the lentivirus-containing supernatant was added dropwise to 1.5 × 10^5^ PDAC93 cells pre-conditioned with 8 μg/mL polybrene. After 48 h, the culture medium was replaced with fresh medium containing 5 μg/mL puromycin (ThermoFisher, Waltham, MA, USA, P8833). Antibiotic-resistant cells were then selected using a FACSAria II cell sorting system (BD Biosciences).

### 5.7. Time-Lapse Microscopy and Organoid Seed Tracking

Time-lapse movies of PDAC93-GFP cells embedded in hydrogels were captured using a LSM-880 inverted CLSM (Zeiss, Jena, Germany), equipped with a 10× Plan-Neofluar (0.30 NA) objective and an excitation wavelength of 488 nm. Immediately after hydrogel gelation, Z-stack images covering a volume of 850 × 850 × 150 µm^3^ were captured every 20 min over a 16 h. The early organoid seeds (EOSs) were maintained at 37 °C and 5% CO_2_ throughout the experiment.

3D migration tracks of EOSs were obtained from the processed time-lapse movies using the TrackMate v6.0.3 single-particle tracking plugin for FIJI v2.16.0 (Bethesda, MD, USA) [81]. The particle-tracking parameters used for 3D EOS trajectory reconstruction are summarized in Table S2. The mean speed (µm/h) (Equation (1)) was calculated using a custom Python v. 3.13.5 script. Additionally, the directness index was calculated by dividing the Euclidean distance (µm) by the accumulated distance (µm) between the starting and endpoint of a migrating EOS. A directness value close to 0 indicates indirect migration, while a value close to 1 suggests straight-line migration.

(1) $$\;v=\frac{1}{N}\sum_{i=1}^{N}\frac{\sqrt{{\left(x_{i}-x_{i-1}\right)}^{2}+{\left(y_{i}-y_{i-1}\right)}^{2}+{\left(z_{i}-z_{i-1}\right)}^{2}}}{\left(t_{i}-t_{i-1}\right)}$$

Protrusions from EOSs were analyzed using a custom Fiji v2.16.0 (Bethesda, MD, USA) script. Time-lapse movies were processed using the CLIJ2 v2.5.3.1 package for FIJI v2.16.0 (Bethesda, MD, USA) [82]. First, 3D videos were transformed into 2D time-series images using a maximum intensity projection (MIP), followed by Laplacian of Gaussian (LoG) filtering for edge detection. EOSs were then segmented using the Weka Segmentation plugin for FIJI v2.16.0 (Bethesda, MD, USA) [83], generating a binary mask. Binary morphological opening was performed to remove cell protrusions, and the XOR logical operator was applied to obtain the protrusion mask. The number and length of segmented protrusions were analyzed using MorpholibJ v1.6.4 [84].

### 5.8. Organoid Morphology Classification

On day 7, mature PDAC93-GFP organoids were fixed with 4% paraformaldehyde (PFA) at 37 °C for 30 min, washed thoroughly, and stained with the SiR-DNA far-red labeling probe (Spirochrome, Stein am Rhein, Switzerland, SC007) for 1 h at room temperature (RT). Mature organoids imaging was performed using a Zeiss LSM-880 AxioObserver inverted confocal laser scanning microscope (CLSM) equipped with a 25× LD LCI Plan-Apochromat (0.8 NA, W) objective. Image stacks covering a total volume of 1065 × 1065 × 200 μm^3^ were acquired using a Mai-Tai^®^ DeepSee™ Ti-Sapphire laser, with sequential scanning at 740 nm for nuclei and 920 nm for cytoplasmic GFP. Prior to quantification, nuclei, and cytoplasmic were segmented using StarDist 3D [85] and the Weka Segmentation 3D plugin [83], respectively. The StarDist 3D v0.9.1 model was trained from scratch for 50 epochs on 45 paired image patches (patch size: 72 × 72 × 32, batch size: 1, number of rays: 32, with augmentation enabled), utilizing an NVIDIA Quadro P1000 GPU on an Intel^®^ Core™ i7-8700K CPU 3.70GHz with 64GB RAM (Windows 10 Pro). Organoid segmentation masks from the obtained Z-stacks were analyzed using a homemade Fiji v2.16.0 (Bethesda, MD, USA) script. In brief, cytoplasmic masks were pre-processed using a GPU-accelerated 3D median filter and binary closing, available in the CLIJ2 v2.5.3.1 library [82]. Each organoid was labeled, and the intersection of the cytoplasmic and nuclear masks was computed using a logical AND operator. Morphological descriptors were quantified using MorpholibJ v1.6.4 (see Table S3).

The classification of mature organoids was performed using a support vector machine (SVM) learning algorithm implemented in the Scikit-Learn Python library v1.7.1 [86]. The SVM model differentiated organoid phenotypes based on the extracted morphological descriptors mentioned above. The SVM structure employed a radial basis function (RBF) kernel, with the regularization parameter set to C = 1 and the γ value internally calculated by Scikit-Learn v1.7.1. Features were standardized using Z-score normalization, and the most relevant morphological descriptors were selected based on their highest κ-statistic scores (see Table S3) for dimensionality reduction. Two separate SVM models were trained for different media conditions (regular and TGFβ), using an independent expert-annotated dataset. The SVM model for regular-conditioned media classified organoids into three categories: Cyst, Solid, and Invasive. The SVM model for TGFβ-conditioned media functioned as a binary classifier, distinguishing between Solid and Invasive morphologies. The decision to use separate SVMs was based on observed morphological differences between each condition prior to organoid annotation, as cystic structures were absent under TGFβ treatment. Model validation via 10-fold cross-validation achieved accuracies of 95% for regular media and 100% for TGFβ-conditioned media.

To visualize mature organoid morphology distributions between regular and TGFβ-conditioned media, t-distributed stochastic neighbor embedding (t-SNE) plots were generated using the openTSNE v1.0.2 library [87]. Specifically, two separate t-SNE models were created from the result of each SVM classifier for organoids from each media conditioning. A third t-SNE model combined results from both SVM classifiers to illustrate overall morphological distributions across conditions, regardless of the cultured media used. Each t-SNE model ran for 1000 iterations with a perplexity of 50, using a Euclidean metric to compute distances between morphological descriptors after organoid classification.

### 5.9. Real-Time Quantitative PCR (RT-qPCR)

Total RNA was extracted on day 7 from mature PDAC93 organoids using the Maxwell RSC SimplyRNA Kit (Promega, Madison, WIS, USA, AS1390) following the manufacturer’s protocol. Complementary DNA was synthesized using the M-MLV Reverse Transcription Kit (Waltham, MA, USA,, 28025013). Quantitative real-time PCR was performed using SYBR Green Master Mix (ThermoFisher, Waltham, MA, USA, A46109) on a QuantStudio5 real-time system (ThermoFisher, Waltham, MA, USA). Gene expression levels were normalized to the endogenous GAPDH reference gene using the 2^−ΔΔCT^ method [88], calculated as follows: 2^−ΔΔCT^ = [(CT gene of interest−CT housekeeping) Organoids−(CT gene of interest−CT housekeeping) 2D-plated cells]. Primer sequences are listed in Table S4. To visualize the gene expression fingerprint, a hierarchical heat-map was generated using a custom Python script with the Seaborn v0.13.2 library [89]. Gene expression values were converted to Z-scores by: Z = (x_1_ − mean)/sd.

### 5.10. Immunofluorescence Staining

Mature PDAC93 organoids were fixed on day 7 with 4% paraformaldehyde (PFA) at 37 °C for 30 min, then permeabilized with 0.1% Triton-X (Merck, Darmstadt, Germany, T8787) at room temperature (RT) for 15 min. To minimize non-specific binding, samples were blocked with 1% bovine serum albumin (BSA) (Merck, Darmstadt, Germany, A7030) in PBS at RT for 30 min. Organoids were then incubated with specific epithelial-mesenchymal transition (EMT) antibodies: anti-E-cadherin Alexa488-conjugated (BD Biosciences, 560061), anti β-catenin PE-conjugated (R&D Systems, IC13292P) and anti-vimentin Alexa647-conjugated (ThermoFisher, Waltham, MA, USA, MA5-11883) at 4 °C for 48 h. For live mitochondria labeling, organoids were incubated in regular media containing 5 μmol/L MitoTracker™ Green probe (ThermoFisher, Waltham, MA, USA, M7514) at 37 °C for 20 min. Z-stack confocal laser scanning microscopy (CLSM) images were acquired using a Zeiss LSM-800 inverted CLSM, equipped with a 40× Plan-Apochromat (1.2 NA, W) objective. Excitation wavelengths were adjusted according to the spectral properties of the conjugated antibodies.

### 5.11. Collagen-I Remodeling Analysis

For PDAC93-induced Collagen-I remodeling, PDAC93 organoids were cultured as described in the Section 5.5. On day 7, mature organoids were fixed with 4% paraformaldehyde (PFA) at 37 °C for 30 min. Cells were then counterstained with SiR-DNA and rhodamine-phalloidin (Abcam, Cambridge, UK, ab235138) for F-actin filament visualization, following the manufacturer’s protocols. Collagen-I remodeling was imaged as described above. Fiber anisotropy (α) and compaction were analyzed using the method previously described by our group [25].

### 5.12. Mitochondrial Bioenergetics Assessment

Real-time measurements of oxygen consumption rate (OCR) and extracellular acidification rate (ECAR) in mature PDAC93 organoids (Day 7) were conducted using the Seahorse XFp Extracellular Flux Analyzer (Agilent Technologies, Santa Clara, CA, USA). Organoids were harvested from the micro-device and resuspended in unbuffered XF-DMEM media (Agilent Technologies, 103575-100) supplemented with 25 mM glucose, 4 mM glutamine, and 1 mM pyruvate. The suspension was then plated onto poly-D-lysine-pre-coated XFp well plates (Seahorse Bioscience, Billerica, MA, USA). OCR and ECAR were measured before and after the sequential addition of metabolic modulators: 0.5 μmol/L oligomycin, 1 μmol/L FCCP, 1 μmol/L rotenone, 1 μmol/L antimycin A, and 100 mM 2-deoxy-D-glucose (2-DG) (all from Merck, Darmstadt, Germany: O-4876, C-2920, A-8674, R-8875, D8375), following the manufacturer’s protocol. Data were normalized to protein content using RIPA lysis and extraction buffer (Thermo Fisher, Waltham, MA, USA, 89901) and quantified using a BCA Protein Assay Kit (Thermo Fisher, Waltham, MA, USA, 23225). Spare respiratory capacity (SRC) and glycolytic capacity (GC) were calculated as the difference between their maximum and basal values. Mitochondrial and glycolytic ATP production rates were determined as described by Desousa et al. [90].

### 5.13. Flow Cytometry Analysis

Mitochondrial membrane potential (Δψm) in mature PDAC93 organoids was assessed by flow cytometry using the MitoProbe-TMRM Assay Kit (Thermo Fisher, Waltham, MA, USA, M20036). On Day 7, mature organoids were dissociated into single cells and incubated in fresh media containing 125 ng/mL TMRM dye for 20 min at 37 °C to stain live mitochondria. After thorough washing, samples were analyzed using a CytoFLEX LX system (Beckman Coulter, Brea, CA, USA). Flow cytometry data were processed using FlowJo™ software (v10, FlowJo, Ashland, OR, USA).

### 5.14. Cytotoxicity Assays

The sensitivity of PDAC93 organoids to Gem (MedChem, USA, Monmouth Junction, NJ HY-B0003) was evaluated using a Live/Dead™ Viability/Cytotoxicity Kit (Thermo Fisher, Waltham, MA, USA, L3224). After five days of incubation, the culture medium was replaced with 100 µL of Gem solution at a final concentration of 0.1 µM, followed by a 48-h treatment at 37 °C and 5% CO_2_. On Day 7, organoids were rinsed, and Syto9 (green, live) and propidium iodide (PI) (red, dead) were added at final concentrations of 7 µM and 40 µM, respectively, followed by a 2-h incubation. The IG_50_ value for Gem was determined using PDAC93 organoids embedded in Matrigel and cultured in regular media with Gem concentrations of 0, 0.001, 0.1, 1, 10, 100, and 1000 µM.

Organoids were imaged using a Zeiss Cell Observer Spinning Disk microscope with a 25× LD LCI Plan-Apochromat (0.8 NA, W) objective. At least 10 images, covering a total sample volume of approximately 607 × 607 × 150 µm^3^, were acquired. Image acquisition was performed using an excitation wavelength of 488 nm for Syto9 (live) and 561 nm for PI (dead). Viability was quantified using a custom script in Fiji v2.16.0 (Bethesda, MD, USA). Syto9 and PI signals were segmented separately using fixed-intensity threshold values. The PI and Syto9 masks were intersected using the logical AND operator to generate a live organoid mask. Relative viability was calculated as the ratio of live organoids in Gem-treated versus regular media conditions.

### 5.15. Mice and Subcutaneous PDAC93 Syngeneic Murine Model

Male C57Bl/6J mice (5–6 weeks old) were obtained from Charles River Laboratories (Strain Code: 027) and housed in a pathogen-free facility at the CIMA Animal Facility, University of Navarra (registration number ES31-2010000132). For tumor implantation, PDAC93 organoids were cultured as described in the Section 5.5 and subcutaneously implanted into the lower right flank on day 7, preserving the embedding hydrogel (M, CM, and C). For drug intervention, once tumors reached 50 mm^3^ (measured via echography on day 14), mice were randomly assigned to six experimental groups (*n* = 9 per group) and treated with either a vehicle control (0.9% saline, i.p.) or Gemcitabine (Gem) (100 mg/kg, i.p., twice a week for two weeks) (Medchem, HY-B0003). On day 28, tumor volume and vasculature were analyzed using the Vevo3100 ultrasound system (Fujifilm, VisualSonics, Toronto, ON, Canada) with an MX550D transducer. Mice were anesthetized with a 2% isoflurane/air mixture (IsoVet^®^, Piramal, Rouboslaan, The Netherlands, 469860) and positioned on a heated pad. ECG sensors were attached to their limbs for vital sign monitoring. Once tumors were identified, B-mode and color Doppler-mode images were automatically acquired along the tumor length. Tumor volume (adjusted per the μg of organoids inoculated) and vasculature (adjusted per tumor volume) were quantified using VevoLab software (v5.8.1, Fujifilm, VisualSonics). A trained technician semi-automatically delineated tumor margin. Tumor growth inhibition (TGI) was calculated using the formula: TGI = (*Vf* − *Vi*)/*Vi*, where *Vi* and *Vf* represent tumor volumes on days 14 and 28, respectively. A TGI > 0 indicated tumor growth, while a TGI < 0 indicated tumor regression. Subcutaneous tumors were harvested at the experimental endpoint (day 28).

### 5.16. Immunohistochemistry Staining

Following tumor collection, samples were fixed in 4% neutral-buffered formalin for 48 h, dehydrated, and embedded in paraffin. Paraffin-embedded samples were cut at 5 μm thickness and immunostained for GATA6 (Cell Signaling, Danvers, MA, USA, 5851). Hematoxylin and eosin (H&E) staining was performed using standard protocols to assess tumor area. Whole-slide images were acquired at 20× magnification using an Aperio CS2 scanner and analyzed with FIJI v2.16.0 (Bethesda, MD, USA) software. A custom plugin was created to select at least 10 non-overlapping ROIs, each measuring 1000 × 1000 pixels, within the tumor area. The DAB chromogen signal was extracted and segmented using a fixed intensity threshold. GATA6-positive cells were quantified and normalized to the total tissue area within each ROI.

### 5.17. Quantification and Statistical Analysis

Hierarchical clustering of stromal components was performed using RNA-seq data from micro-dissected PDAC tumor stroma [7] and proteomic data from Matrigel GFR and rat tail collagen-I [91]. In brief, we cross-referenced the primary scaffolding proteins identified in each commercial matrix, based on the proportions used in our hydrogels, with those enriched in stroma-specific PDAC subtypes -active and normal. This analysis allowed us to evaluate the correspondence between hydrogel composition and stromal PDAC subtype signatures.

CDH1, CTNNB1 and VIM signal distribution was analyzed using a Fiji v2.16.0 (Bethesda, MD, USA) script adapted from Nyga et al. [92] for Z-stack imaging. A region of interest (ROI) encompassing at least two nuclei was manually selected, and a straight line was drawn between them. Intensity values for each channel were normalized by subtracting the 1st percentile and dividing by the 99th percentile. Junctional CDH1 intensity was extracted by averaging pixel intensities above Otsu’s threshold [93] within the ROI volume, following a logical AND operation between the junctional and cytoplasmic masks. Simultaneously, SiRDNA signal was segmented using Otsu’s threshold, and junctional CTNNB1 intensity was determined via a logical AND operation between the junctional and nuclear masks. The ratio of junctional to cytoplasmic CDH1 intensity was calculated by dividing junctional intensity by either the average cytoplasmic intensity or the average nuclear intensity for the CTNNB1 marker. The cytoplasmic intensity of the VIM channel was determined using Otsu’s threshold method, as described above. The CDH1/VIM ratio was then calculated by dividing the average junctional CDH1 value by the average cytoplasmic VIM value, followed by log transformation. This ratio classified PDAC93 organoids as epithelial (log CDH1/VIM ratio > 0) or mesenchymal (log CDH1/VIM ratio < 0) phenotypes [36].

Mitochondrial morphology -including mass (weighted per cell number), mean volume, sphericity, and branching- was analyzed using the “3D Mitochondria Analyzer” plugin for Fiji v2.16.0 (Bethesda, MD, USA) [94] with default parameters.

Statistical analyses were conducted using GraphPad Prism (version 8, GraphPad, San Diego, CA, USA). Data are presented as mean values, with error bars representing the standard deviation of biological replicates. The normality of data distribution was assessed using the Kolmogorov-Smirnov and Shapiro-Wilk tests. Based on the distribution, appropriate statistical tests were applied: (i) Parametric data, a T-test was used for comparisons between two independent samples. For comparisons involving more than two groups, ANOVA was performed, followed by Tukey’s or Tamhane’s T2 multiple comparison tests, depending on variance homogeneity; (ii) Non-parametric data, a Mann-Whitney test was used for comparisons between two independent samples. For comparisons involving more than two groups, the Kruskal-Wallis test was applied, followed by Dunn’s multiple comparison test. RT-qPCR fingerprint data were analyzed using multivariate analysis of variance (MANOVA), implemented in the statsmodels 0.12.2 statistical package for Python. A *p*-value < 0.05 was considered statistically significant. Significance levels are denoted in figures with asterisks (*, *p* < 0.05; **, *p* < 0.01; ***, *p* < 0.001). Biological replicates are indicated in figure legends.

## Data Availability

All raw and processed data used in this study can be found in the following repositories: https://doi.org/10.5281/zenodo.18756998 and https://doi.org/10.5281/zenodo.18800480. All original code has been deposited at Zenodo at https://doi.org/10.5281/zenodo.15040153. Any additional information required to reanalyze the data reported in this work paper is available from the lead contact upon request.

## Supplementary Materials

The following supporting information can be downloaded at: https://www.mdpi.com/article/10.3390/gels11070562/s1, Figure S1. Morphological analysis of PDAC93 organoids; Figure S2. Clustering based on scaffolding proteins of the hydrogel; Figure S3. IHC analysis of the expression of GATA6; Figure S4. Micro-device for PDAC93 organoid generation; Figure S5. Micro-device mold for PDMS chamber fabrication; Table S1. Culture media composition used for organoids and cell culture; Table S2. TrackMate particle-tracking parameters used for 3D organoid trajectory reconstruction; Table S3. Morphological descriptors used for PDAC93 organoids classification and their κ-statistic scores; and Table S4. Primer list for clinical subtype and EMT markers.
