# Supplementary material for: Tumor Organoids Grown in Mixed-Composition Hydrogels Recapitulate the Plasticity of Pancreatic Cancers
Source: Gels. 2025 Jul 21;11(7):562. doi: 10.3390/gels11070562 (PMC12294231; doi:10.3390/gels11070562)
Supplement: Supplementary file 1 [file gels-11-00562-s001.zip › gels-3758618-supplementary.pdf]

gels

Supplementary material

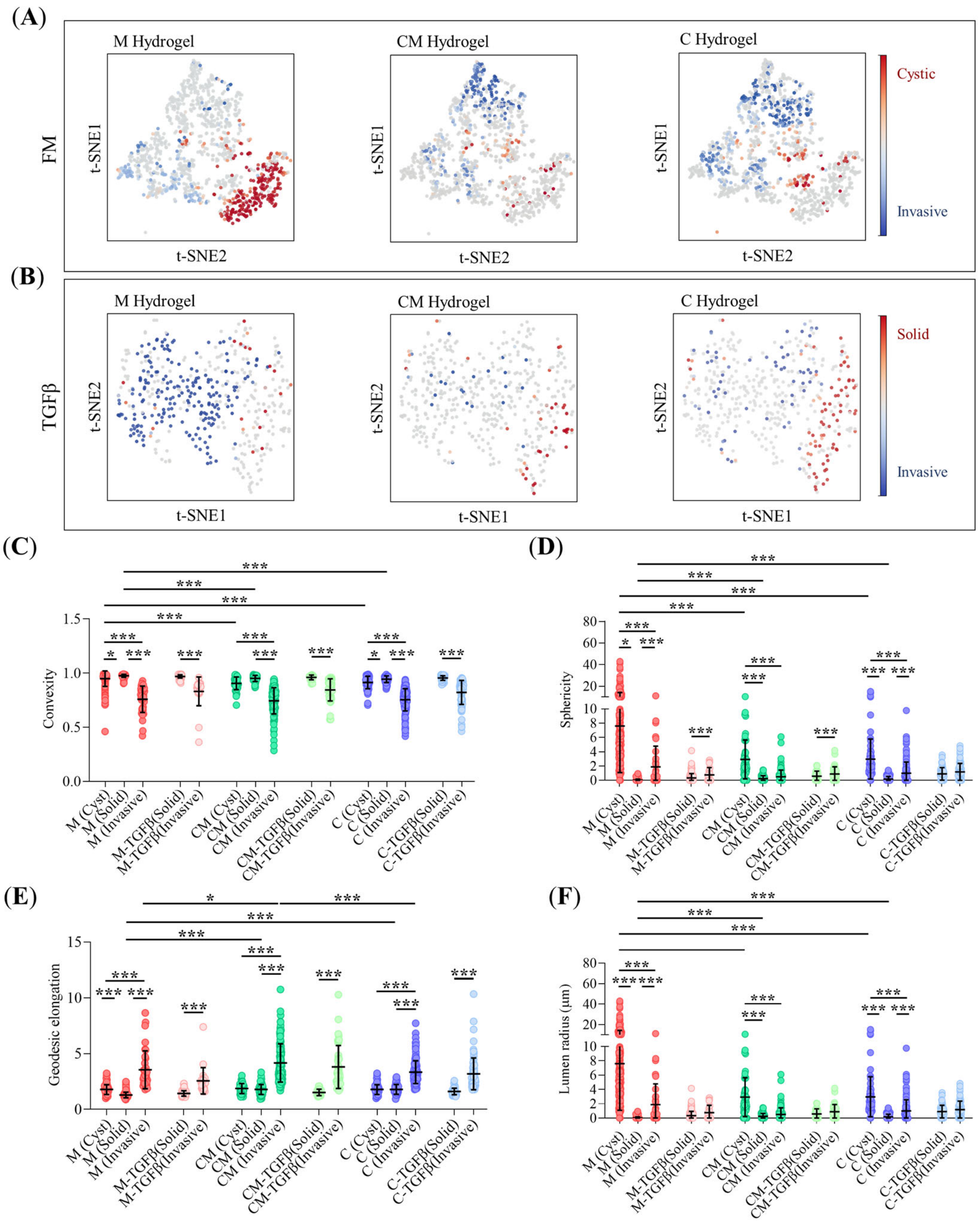

Figure S1. Morphological analysis of PDAC93 organoids. (A and B) t-SNE projections with the morphological classes distribution for PDAC93 organoids under FM and TGFβ conditioning, respectively, categorized by gel type (M, CM, and C hydrogels). Scale bars: probability of classification as Cyst, Solid, or Invasive; (C-F) Quantification of the highest κ-scored morphological descriptors used in the SVM classifier, including organoid convexity, geodesic elongation, and lumen radius, categorized by morphological classes for each gel type and cell media. (*n* = at least 150 organoids from 3 biological replicates). Error bars: mean ± SD; ***, $p<0.01$, *, $p<0.05$. Wilcoxon rank-sum test and Mann-Whitney test (C-F)

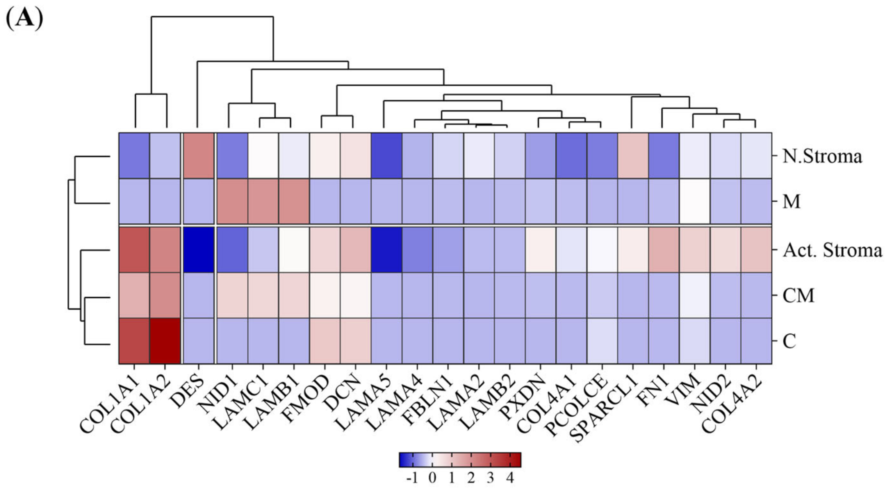

Figure S2. Clustering based on scaffolding proteins of the hydrogel. (A) Hierarchical heat-map showing the expression (Z-score) of major scaffolding proteins expressed in our hydrogels and in "activated" and "normal" stroma (data extracted from: Moffitt, R., Marayati, R., Flate, E. et al. Virtual microdissection identifies distinct tumor- and stroma-specific subtypes of pancreatic ductal adenocarcinoma. Nat Genet 47, 1168–1178 (2015). https://doi.org/10.1038/ng.3398).

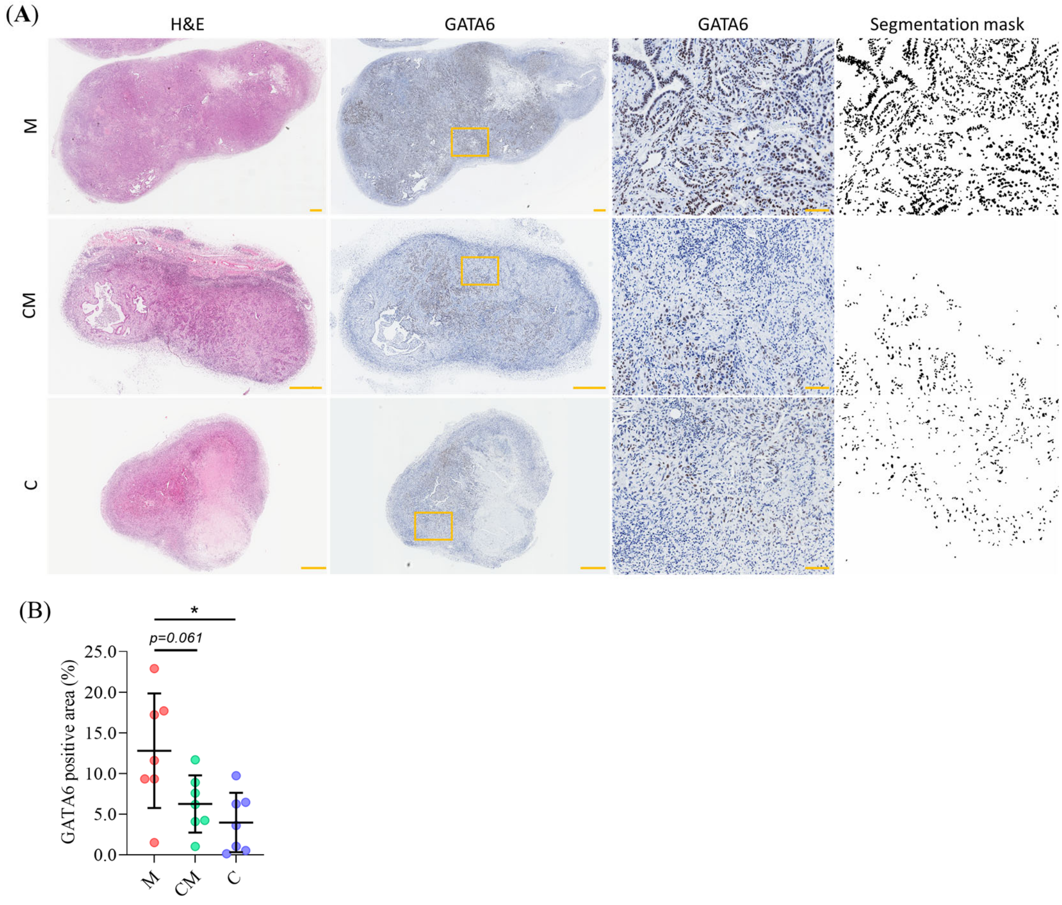

Figure S3. IHC analysis of the expression of GATA6. (A) H&E and GATA6-stained images of subcutaneous tumors collected from mice in different hydrogel groups after sacrifice. Yellow square insets show representative areas with GATA6 staining. The segmentation mask was extracted from the yellow square inset. Scale bars: 500 μm for low-magnification images (left panel) and 100 μm for

high-magnification images (right panel); (B) Quantification of GATA6-positive-stained area. (n = 7 slices). Error bars: mean ± SD; One-way ANOVA (B).

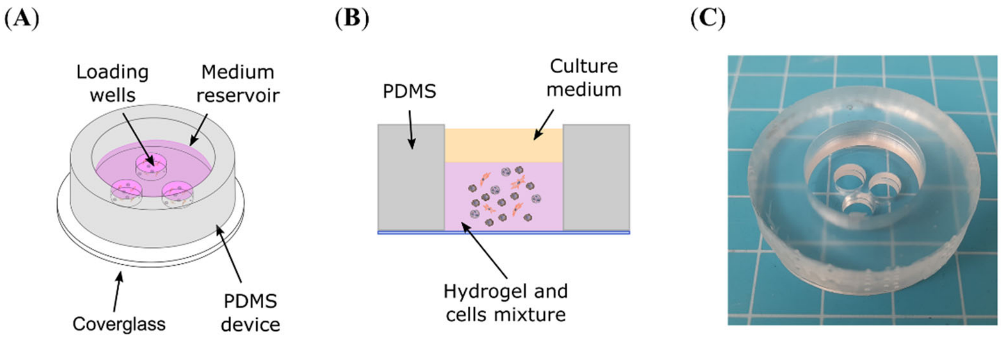

Figure S4. Micro-device for PDAC93 organoid generation. (A) Schematic of the micro-device; (B) Cross-sectional view showing a single well with the hydrogel and embedded organoids; (C) Photograph of the PDMS micro-device.

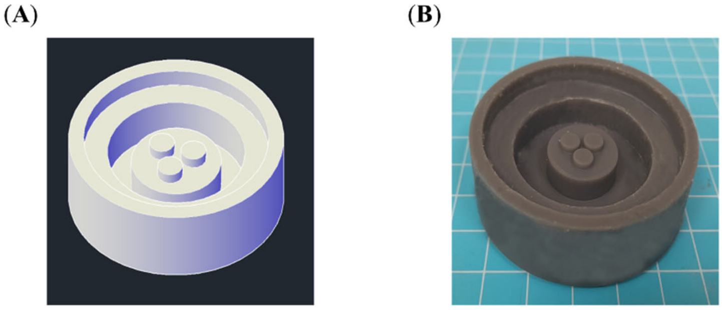

Figure S5. Micro-device mold for PDMS chamber fabrication. (A) 3D CAD model; (B) Photograph of the resin mold printed by stereolithography.

Table S1. Culture media composition used for organoids and cell culture

Reagent	Source	Concentration	Used in
DMEM	Gibco	1X	2D-Cell media
FetalClone-III	Cytiva	10% v/v	2D-Cell media
Advanced DMEM F12	Gibco	1X	Organoid media
Penicillin/Streptomycin	Gibco	1X	2D-Cell/Organoid media
HEPES solution	Corning	1X	Organoid media
GlutaMax	Gibco	1X	Organoid media
A83-01	Merck	0.5μM	Organoid media*
mEGF Recombinant Protein	ThermoFisher	50 ng/mL	Organoid media
mFGF-10 Recombinant Protein	ThermoFisher	100 ng/mL	Organoid media
Gastrin I	Tocris Bioscience	10 nM	Organoid media
mNoggin Recombinant Protein	Peprotech	100 ng/mL	Organoid media*
Y-27632 dihydrochloride	Merck	14 μM	Organoid media

Reagent	Source	Concentration	Used in
DMEM	Gibco	1X	2D-Cell media
N-Acetyl-L-cysteine	Merck	1.25 mM	Organoid media
Nicotinamide	Merck	10 mM	Organoid media
B-27™ Supplement	ThermoFisher	1X	Organoid media
TGF-beta 1 Recombinant Protein	R&D Systems	1 ng/mL	Organoid media

* These growth factors were excluded in the TGFβ-supplemented medium.

Table S2. TrackMate particle-tracking parameters used for 3D organoid trajectory reconstruction

Processing step	Parameter/value
LoG detector	radius = 12.5 μm<br>threshold = 2.5 μm<br>subpixel = True<br>median = True
Simple LAP tracker	max_frame_gap = 0<br>max_distance = 20.0 μm<br>max_gap_distance = 20.0 μm

Table S3. Morphological descriptors used for PDAC93 organoids classification and their κ-statistic scores

Feature	Description	F-score
Circularity:	The normalized ratio of the area over the square of the perimeter ($4\pi \cdot A/p^2$)	180.83
Convexity:	The ratio of the perimeter of the convex hull of the particle to the perimeter of the particle	173.87
Sphericity:	The ratio of the squared volume over the cube of the surface area ($36\pi V^2/S^3$). Normalized so that the value for a perfect sphere equals one.	87.72
Geodesic.Elong:	The ratio of the geodesic diameter to the diameter of the largest inscribed circle	87.47
R.Lumen:	The radius of the largest inscribed sphere for the lumen of an organoid	58.12
R1.Ellipse:	The radius of the major axis of an ellipse	45.59
Ellipse.Elong:	The ratio of the largest over the smallest axis lengths	41.00
Vol Lumen/Vol Total:	The ratio between the total number of voxels comprising the Vol Lumen and Vol Total	39.92
GeodesicDiameter:	The length of the longest geodesic path within a particle	38.64
R1:	The length of the radius 1 of an ellipsoid	34.51
Ball Radius:	The minimum sphere circumscribed in the Vol Total	29.95
InscrDisc.Radius:	The minimum radius inscribed in the Vol Total	28.07
R.Organoid:	The radius of the largest inscribed sphere within an organoid	27.88
R1/R3:	The elongation of the object is defined as the ratio between the length and the width	27.65
R2:	The length of the radius 2 of an ellipsoid	26.48
R3:	The length of the radius 3 of a 3D ellipsoid	26.28

R2.Ellipse:	The radius of the minor axis of an ellipse	25.35
Perimeter:	Length of the boundary comprising a particle, using the Crofton formula.	24.19
Nuclei Number:	The number of nuclei corresponding to the instances obtained from SirDNA Staining	15.89
Surface GFP:	The total area of the uppermost layer of the organoid GFP instance.	12.91
ConvexArea:	The minimum enclosed convex-shape area (convex hull) of a particle	11.59
Organoid Area:	The number of pixels comprising the organoid instance is multiplied by the area of each pixel applied to the maximal projection of the organoid mask.	11.39
Vol GFP:	The number of voxels comprising the GFP staining	10.64
Vol Total:	The total number of voxels obtained by the sum of Vol GFP and Vol Lumen	9.84
R2/R3:	The flatness of the object is defined as the ratio between the width and height	7.08
Vol Lumen:	The number of voxels comprising the organoid lumen	4.69
Tortuosity:	The ratio between the shortest pathway to the distance between the inlet and outlet plane	4.56

Table S4. Primer list for clinical subtype and EMT markers

Gene	Marker-family	Sequence
HNF1a-Fw	Subtype	5′-AGAGACCTTGGTGGAGGAGTGT-3′
HNF1a-Rv	Subtype	5′-GGCAAACCAGTTGTAGACACGC-3′
TSPAN8-Fw	Subtype	5′-AGGTTTCCTGGGATGCTGTGGA-3′
TSPAN8-Rv	Subtype	5′-ACTCAGGTTTGAAAGCGGCTCC-3′
CDH1-Fw	EMT	5′-AGCGGCAAGAGTGAGATTTCT-3′
CDH1-Rv	EMT	5′-CCTCCAGGTTATTCTCCAGGG-3′
CDH2-Fw	EMT	5′-TGAAACGGCGGGATAAAGAG-3′
CDH2-Rv	EMT	5′-GGCTCCACAGTATCTGGTTG-3′
VIM-Fw	EMT	5′-CAAGAGCGCCTTGACGATACA-3′
VIM-Rv	EMT	5′-CCAAGAGACAGGTTTCTCCATC-3′
ZEB1-Fw	EMT	5′-CTCTGCAAGAGACTTCCATCCAGT-3′
ZEB1-Rv	EMT	5′-GAAGTAGGGAAGGCCGTGG-3′
GAPDH-Fw	Endogenous	5′-ACTTTGTCAAGCTCATTTCC-3′
GAPDH-Rv	Endogenous	5′-TGCAGCGAACTTTATTGATG-3′
